# LLMs for industrial databases: an agro-food production plant use case

**DOI:** 10.3389/frai.2026.1764367

**Published:** 2026-05-29

**Authors:** Lacramioara Dranca, Pablo Donate, Julio A. Sanguesa, Piedad Garrido, Vicente Torres-Sanz, Francisco J. Martinez

**Affiliations:** 1 Centro Universitario de la Defensa (CUD), Zaragoza, Spain; 2 Department of Computer Science and System Engineering, University of Zaragoza, Teruel, Spain

**Keywords:** industrial energy management, knowledge graph, large language models, natural language interfaces, NL-to-InfluxDB queries, small language models, time series databases

## Abstract

**Introduction:**

Extracting value from industrial time-series databases such as InfluxDB 2.0 requires expertise in specialized query languages (InfluxQL, Flux) that domain experts typically lack, and no labeled corpora exist for translating natural language into them.

**Methods:**

We present a modular system that translates questions written in Spanish into executable InfluxQL and Flux queries over an InfluxDB 2.0 instance deployed in a real agro-food production plant operating under a self-consumption energy scheme. It comprises a semantic layer implemented as a knowledge graph encoding the InfluxDB schema and its correspondence with the plant's physical components; a hierarchical entity-linking module; a lightweight language model fine-tuned for NL-to-query generation; and a query validation and sanitization module. To obtain training data, we develop a fully automated synthetic dataset distillation pipeline that uses a large teacher model with contextual retrieval from the official InfluxDB 2.0 documentation; each candidate undergoes parsing, AST extraction, and semantic checking against the knowledge graph, and only validated samples are retained. The corpus fine-tunes compact Small Language Models through domain-level conditioning followed by task-specific instruction tuning.

**Results:**

Performance is evaluated on a manually curated suite of 56 Spanish queries, evenly distributed across seven operationally relevant query families. Compact models reliably generate syntactically valid queries, but functional evaluation against the live instance reveals a three-layer pattern-high parser validity, moderate execution success, and lower result correctness-that locates the residual gap at the semantic layer.

**Discussion:**

The contributions are a complete NL-to-InfluxDB pipeline grounded in an explicit semantic representation of a real industrial schema; a documentation-driven synthetic data generation process with automatic syntactic and semantic verification; and a parameter-efficient fine-tuning strategy enabling query generation with lightweight models suitable for resource-constrained environments.

## 1 Introduction

The digitalization of industry, within the framework of Industry 4.0, has triggered an unprecedented explosion in the generation of time-series data. Cyber-Physical Systems production plants, and particularly modern energy infrastructures, continuously monitor their operations. This generates massive flows of high-frequency, high-volume data, where the speed of analysis is critical (Jensen et al., 2017). This data is the cornerstone of Critical Decision Support Systems, especially in sectors like agri-food, where operational margins are tight, and energy management (in self-consumption models) is crucial. Its analysis allows for optimizing energy efficiency, predicting machinery failures, and improving resource management in real-time.

However, this wealth of data presents a paradox: the ability to extract value from it remains largely limited to users with a high technical profile. Time-Series Databases (TSDBs), such as InfluxDB, Prometheus, or TimescaleDB, have evolved to efficiently manage this workload, but have done so by developing specialized query languages (e.g., InfluxQL, Flux, and PromQL). These languages, often functional and stream-based, introduce a query paradigm significantly different from declarative SQL. This specialization creates a usability gap, preventing domain experts (plant engineers, energy operators) from directly interacting with the information they need (Katsogiannis-Meimarakis and Koutrika, 2023).

To bridge this gap, research has focused on Natural Language Interfaces (NLIs). The rise of Large Language Models (LLMs) has led to notable advances in Text-to-SQL translation (Li et al., 2023). However, these advances are not directly transferable to the time-series domain. The query semantics differ substantially; SQL relational algebra lacks the native abstractions for time windowing, downsampling, complex temporal aggregations, or stream processing, which are central to TSDBs (Baek et al., 2023). Attempts to apply standard Text-to-SQL models to time-series queries fail due to this fundamental data model mismatch.

Addressing the “Text-to-Time-Series-Query” problem requires overcoming two fundamental challenges. The first is the critical scarcity of training corpora. Unlike SQL, where Spider dataset (Yu et al., 2018) is a notable example, there are no large-scale, labeled datasets that map natural language questions to queries in InfluxQL or Flux (the query languages of InfluxDB). Manually creating such a corpus is prohibitively expensive, as it requires “dual expertise”: a deep understanding of the industrial domain and high technical proficiency in TSDB query languages.

The second challenge is the inherent semantic ambiguity, known as the “semantic grounding” problem. Users formulate questions using domain vocabulary (e.g., “milking machine consumption” or “tank room temperature”). The system must unambiguously map these concepts from the user's “mental model” to the physical database structures (e.g., measurements, tags, and fields). LLMs (Large Language Models), by themselves, struggle with this mapping when the schema is complex, or the terminology is plant-specific (Cheng et al., 2022).

This paper presents a complete architecture for natural language interaction with industrial databases, applied to a real-world use case in an agri-food production plant. To overcome the data scarcity challenge, we propose a fully automated pipeline that generates a high-quality synthetic dataset. This process uses Knowledge Distillation, employing LLMs (*teacher* models) to extract (NL, Query) pairs directly from the official InfluxDB technical documentation. We view the documentation as an untapped corpus that already contains semantic intent (NL descriptions) linked to code examples (Queries). In line with this idea, domain-specific synthetic data generation via self-instruction has proven to be an effective strategy for enhancing specialized query tasks (Wang et al., 2023). Next, the distilled knowledge is used for fine-tuning smaller, more efficient models (SLMs - Small Language Models), optimizing the trade-off between accuracy and computational cost.

However, query generation by the SLM alone can suffer from semantic ambiguity and loss of factual grounding. To address this limitation, we integrate a Knowledge Graph (KG) that acts as an explicit intermediate semantic layer. Instead of relying solely on the implicit context in the SLM's prompt, the KG provides factual, structured, and queryable knowledge about the plant's assets, their relationships, and their mapping to the TSDB schema. This synergy, where the KG grounds the queries that the SLM generates, is an active research line for mitigating LLMs “hallucinations” and improving their accuracy in factual domains (Pan et al., 2024).

The main contributions of this work are:

- A complete NL-to-InfluxDB architecture that integrates a small language model, a knowledge graph, and automated syntactic and semantic verification.
- A fully automated documentation-driven distillation pipeline that generates high-quality synthetic NL–to–Flux/InfluxQL pairs without human labeling. The pipeline combines large language models with multi-layer validation (parsing, syntactic, and semantic consistency checking against the knowledge graph), ensuring that only syntactically and semantically valid queries are used for fine-tuning.
- A parameter-efficient fine-tuning strategy that enables query generation with lightweight models suitable for resource-constrained environments.
- The evaluation of the architecture in a real-world industrial energy scenario using a manually created evaluation dataset that spans both Flux and InfluxQL queries.

The industrial case study presented in this work is deployed on InfluxDB 2.x, targeting both Flux and InfluxQL as practical, deployment-relevant query languages. This choice reflects the installed base reality of production time-series deployments rather than latest-version availability. InfluxDB remains the most widely adopted open-source TSDB in both academic benchmarks and production environments (Bader et al., 2017; Liu and Yuan, 2019; Shah et al., 2022), but has undergone three major architectural rewrites (1.x → 2.x → 3.x), each introducing breaking changes with limited backward compatibility. While InfluxDB 3.x represents the latest development branch, the transition path from legacy deployments is non-trivial: InfluxDB 3 Core (the free tier) is architected as an edge data collector with query time range restrictions and is explicitly not intended as a drop-in replacement for InfluxDB 1.x/2.x installations (InfluxData, 2025c,d). Full-featured migration requires the commercial Enterprise edition. Consequently, large installed bases of InfluxDB 2.x deployments remain operational and are expected to persist for the foreseeable future, motivating our focus on Flux and InfluxQL as practical query targets for real-world industrial systems rather than pursuing support for the latest but commercially restricted version.

The remainder of this article is organized as follows. Section 2 reviews related work in the field of natural language interfaces for databases and synthetic data generation. Section 3 details the materials and methods, including a description of the industrial case study and user query requirements, the proposed system architecture, and model development. Next, Section 4 presents the experimental results of our architecture and an ablation study to evaluate the impact of each component. Finally, Section 5 outlines the conclusions of this work and future research directions.

## 2 Related work

The research landscape relevant to this work can be organized around three central themes: (i) the absence of training corpora for natural language interaction with time-series databases, (ii) the need for explicit semantic grounding to map domain terminology to database structures, and (iii) natural language interfaces for domain-specific, non-SQL query languages.

Recent surveys of LLM-based database interfaces (Hong et al., 2025) further document substantial progress in text-to-SQL for relational databases, with methods organized into in-context learning and fine-tuning paradigms achieving strong performance on benchmarks like Spider and BIRD. However, these advances focus exclusively on SQL-like declarative query languages, leaving time-series-specific paradigms underexplored. The NL-to-TSDB setting is better understood within the broader context of LLMs for low-resource and domain-specific programming languages (DSLs) (Joel et al., 2024).

Furthermore, the need to generate queries across multiple structurally distinct paradigms—functional (Flux) with temporal operators and declarative (InfluxQL)—introduces challenges analogous to polyglot persistence (de Curtó et al., 2025), where LLMs must translate natural language into heterogeneous query languages optimized for different data access patterns. While polyglot persistence typically addresses multi-database architectures (e.g., MongoDB for documents, Neo4j for graphs, Redis for caching), the dual-paradigm requirement in InfluxDB query generation—spanning functional pipeline composition (Flux) and SQL-like aggregation (InfluxQL)—presents analogous translation challenges that extend beyond single-language text-to-SQL approaches. Moreover, whereas text-to-SQL research primarily employs large proprietary models via in-context learning (Hong et al., 2025), our work focuses on compact fine-tuned models (sub-4B parameters) suitable for edge deployment in industrial time-series environments.

### 2.1 Synthetic data generation and knowledge distillation

Labeled corpora for NL-to-query translation are nonexistent for InfluxQL and Flux. This mirrors the challenge faced in low-resource DSL settings such as Verilog, and specialized configuration languages (Joel et al., 2024).

To address similar issues in other domains, prior research has explored synthetic data generation driven by large language models. Frameworks such as SELF-INSTRUCT (Wang et al., 2023) and GAL (He et al., 2022) demonstrate that synthetic corpora, when combined with systematic filtering and validation, can replace human-annotated datasets and substantially improve downstream task performance. Knowledge distillation allows compact “student” models to incorporate domain-specific constraints from more capable teacher models (Hinton et al., 2015; Magister et al., 2023), a key pattern observed in DSL research (Joel et al., 2024).

However, existing work focuses primarily on general instruction-following or SQL-oriented tasks. No prior work generates synthetic NL → query pairs for InfluxQL or Flux, and no study integrates documentation-driven generation with multi-layer validation (parsing, AST -Abstract Syntax Tree- extraction, and schema-aware semantic checking). Our work fills this gap by introducing a fully automated pipeline that distills (NL, Query) pairs directly from the InfluxDB 2.0 documentation. The synthetic queries are validated syntactically and semantically, and at inference time, the SLM's generated queries are additionally validated against the concrete schema of an industrial TSDB.

### 2.2 Semantic grounding and knowledge-graph-assisted query generation

A second line of related work concerns the “semantic grounding” problem: mapping user vocabulary to database entities. Research in Text-to-SQL has shown that schema linking and structured context are critical for accurate translation. Recent systems such as BASE-SQL (Sheng et al., 2025) and DIN-SQL (Pourreza and Rafiei, 2024) explicitly integrate schema information into the generation process. A key insight from Sequeda et al. (2024) is that incorporating a KG can dramatically boost accuracy in enterprise scenarios with complex schemas, underscoring the importance of explicit grounding for LLM-based interfaces.

Beyond relational databases, KG-enhanced prompting has been explored as a strategy to reduce hallucinations and improve factual consistency in LLMs (Pan et al., 2024). Nevertheless, these methods have not been applied to time-series databases, whose abstractions (buckets, measurements, fields, and tags) and temporal semantics differ significantly from relational models.

Our work extends this line of research by employing a KG that encodes both the structure of an InfluxDB 2.0 schema and its correspondence with the physical assets of an industrial plant. This enables precise grounding of user intent and supports semantic validation of generated queries, capabilities that have not been explored in Text-to-Flux/InfluxQL.

### 2.3 Natural language interfaces for domain-specific query languages

The field of natural language interfaces for databases has advanced rapidly, particularly in Text-to-SQL supported by benchmarks such as Spider (Yu et al., 2018) and BIRD (Li et al., 2023). Large-scale benchmarks like BIRD demonstrate that LLMs can handle complex schemas and cross-domain queries in SQL, though these focus exclusively on relational databases and leave time-series-specific query languages underexplored. More recently, research has expanded to domain-specific languages such as PromQL for monitoring (Zhang et al., 2025), demonstrating growing interest in NL interfaces beyond SQL.

Despite these advances, no NL interface has been developed for InfluxQL or Flux, nor do benchmarks exist for time-series query languages. This situation parallels the broader DSL landscape, where researchers must curate specialized evaluation sets supported by domain knowledge (Joel et al., 2024).

Our work contributes the first NL-to-InfluxDB architecture tested in a realistic industrial setting. It introduces a curated benchmark reflecting operational energy-analysis scenarios, positioning NL-to-TSDB as an emerging DSL problem.

## 3 Materials and methods

This section details the materials and methodological procedures used to enable natural language querying of time-series data from an agro-food production plant operating under a self-consumption energy model. The target system transforms natural language questions into executable InfluxQL or Flux queries over InfluxDB 2.0 and returns interpretable results to support operational decision-making.

The methodology rests on three principles. First, a modular design that separates user-intent capture, semantic representation of the domain, and query generation/execution, in order to promote traceability, maintainability, and independent component validation. Second, explicit semantic grounding via a knowledge graph that models the database structure (buckets, measurements, fields, and tags) and its correspondence with the plant's physical elements, reducing lexical ambiguity and ensuring alignment with the actual schema. Third, a fully automated synthetic-data generation pipeline based on distillation from technical documentation, which produces question–query pairs and applies grammatical and semantic verification before incorporating them into the training set.

Within this framework, the content progresses from general to specific: the industrial case study and its query needs are described; the system architecture and the three-stage process (NL, query, and result) flow are presented; the construction of the synthetic dataset and the fine-tuning of lightweight models for query generation are documented; and the evaluation methodology is outlined, including criteria of accuracy, semantic coherence, and computational cost. Sufficient detail on configurations, parameters, and protocols is provided to facilitate reproducibility of the results.

### 3.1 Case study and user query requirements

This subsection presents the case study and specifies the query requirements that guide the remainder of the work. We first describe the plant's electrical and monitoring infrastructure and how it is materialized in a time-series database, and then connect this representation to the requirements of natural language querying.

#### 3.1.1 Case study description

The plant under study is a cheese factory operating under a self-consumption energy scheme. Its electrical topology corresponds to an industrial microgrid with three main subsystems: photovoltaic generation, electrochemical storage, and grid interconnection for both export of surplus energy and supply backup. Centralized control and monitoring are performed through the vendor's remote management environment for the power system, which integrates three-phase metering, battery state of charge (SOC), inverter and charger set points and power limits, and aggregated consumption and production metrics. Complementary machine-level sub-metering uses non-invasive clamp sensors installed on individual feeders, producing per-asset energy measurements together with device-identification metadata.

From an operational perspective, the installation records and analyzes energy transfers along all relevant paths in the microgrid: from solar generation to the plant load, to the battery, and to the public grid; from the battery back to the load and to the grid; and from the grid to the local consumption and to the battery. In addition to these energy flows, the system maintains separate time series for total plant demand and total photovoltaic production. Hourly prediction series are available for both consumption and solar generation, together with forecasts of solar irradiance that characterize the expected resource availability. All signals are stored in the Europe/Madrid time zone, and the prediction series is refreshed daily at midnight to support next-day planning and decision making.

InfluxDB 2.0 is used as the time-series database in this plant, not as a design choice of this work, but as a constraint imposed by the existing industrial infrastructure. Consequently, our methodology adapts to the native abstractions and query languages of this deployment.

Details of the schema are essential because semantic grounding and validation depend directly on these constructs. Minimal abstraction would lose the correspondence constraints modeled in the KG. For the purposes of this work, the following concepts are relevant:

- A point is the atomic write unit, defined by a timestamp, a measurement name, a set of tags represented as indexed textual key–value pairs, and a set of fields that store measured or computed values of numerical or boolean type.
- A measurement is a logical collection of points representing the same phenomenon, such as hourly aggregates or 15-min real-time readings for a given subsystem.
- Tags vs. fields: tags enable efficient filtering and grouping and are typically used to encode discrete attributes such as device identifiers or phase labels, whereas fields carry the numerical values that are the primary subject of analysis. This separation is essential to control schema cardinality and query performance.
- A bucket is a storage namespace with a retention policy and shard-group settings that determine the life cycle and physical organization of the data and group together related measurements.
- Ingestion follows the InfluxDB line protocol, where each point is written as a combination of measurement identifier, tag set, field set, and timestamp.
- Two query languages are used: InfluxQL, which resembles SQL and is convenient for many aggregation and filtering operations, and Flux, which provides functional pipelines well suited for joins, mapping, windowed aggregation, and complex derived computations. Flux query results typically include reserved columns such as time bounds and value columns that facilitate window auditing and aggregation traceability.

At the database level, the information captured in the plant is organized around two main groups of time series, aligned with plant-level and machine-level analysis. The plant-level group stores variables that describe the global behavior of the microgrid. One dataset aggregates energy quantities at an hourly resolution. It includes the total electrical demand of the factory, the energy produced by the photovoltaic array, and the energy exchanged along each path between the main components of the system: energy transferred from solar generation to on-site consumption, to the battery, and to the grid; energy taken from the grid to supply the load and to charge the battery; and energy delivered by the battery to support the load or to inject into the grid. These hourly series make it possible to reconstruct full energy balances, compute self-consumption and export ratios, and evaluate the contribution of storage to peak shaving and arbitrage. The same dataset records the state of charge of the battery at a coarser temporal resolution, together with the minimum and maximum thresholds used by the control logic to protect the storage system.

The hourly plant-level dataset also contains the predictive component of the monitoring system. For each day, it stores forecasts of plant consumption and photovoltaic production at an hourly resolution, as well as expected solar irradiance profiles. These forecast series are generated once per day and remain fixed until the next update at midnight. They enable ex ante analyses such as estimating the fraction of demand that can be covered by solar generation in the upcoming day, identifying likely periods of surplus energy, or anticipating when grid import will be necessary.

A second plant-level dataset captures higher-frequency electrical variables at a resolution of 15 min. It includes instantaneous active power on each phase and in total, phase voltages and currents, and cumulative energy counters. These measurements are complemented with variables that describe the instantaneous operating point of the microgrid controller, such as the current state of charge, the net power exchanged with the grid, the power produced by the photovoltaic system, the power demanded by the loads, and the power flowing into or out of the battery. Additional series indicate the configured maximum power capacity and the power limits imposed on the inverter–charger at each time. This higher-frequency view is particularly useful for diagnosing operational issues, analyzing phase imbalances, and validating that control actions respect contractual limits and equipment ratings.

The second main group of time series in the database is devoted to machine-level sub-metering. It stores hourly energy consumption for each metered device in the factory, as derived from the clamp sensors. Each time series is associated with a small set of textual attributes that identify the physical asset, combining a unique device code with a human-readable equipment name and auxiliary communication parameters. This structure supports indicators such as energy consumption per machine and per shift, rankings of the most energy-intensive assets over a given period, and comparisons between similar pieces of equipment operating under different conditions.

Taken together, these plant-level and machine-level datasets provide a rich description of the electrical behavior of the factory at multiple temporal scales. The numerical time series support a wide range of aggregations and derived indicators, including self-consumption rate, export ratio, battery utilization patterns, and hourly photovoltaic performance. The associated attributes enable segmentation by machine, phase, or subsystem and comparative analysis across devices and time windows. At the same time, the schema defines a stable semantic space for natural language interaction: the terms used by operators in their daily work, such as consumption, production, state of charge, grid import, grid export, or consumption by machine, have a direct and systematic correspondence with specific groups of time series and attributes in the database. This alignment is essential for the subsequent components of the system, which must translate informal questions into executable queries without requiring users to know the internal structure or naming conventions of the database.

#### 3.1.2 User query requirements

The proposed system is framed within a software context that incorporates machine learning components, where the definition of requirements and, in particular, the construction of test suites cannot strictly follow classical development patterns. As noted by Wan et al. (2019), the development of ML-based systems introduces additional uncertainty in both requirements and quality, and demands greater effort in the creation of test sets that are specific and representative of real use cases. In our case, this translates into explicitly designing which types of natural language queries must be supported and how we will evaluate, in a reproducible manner, the system's ability to generate correct InfluxQL and Flux queries.

Following the idea that evaluation datasets must be constructed deliberately and carefully, rather than as an accidental by-product of system development, we adopt an adaptation of the dataset creation workflow described by Orr and Crawford (2024): definition of parameters and target population, design of the collection procedure (in our case, expert manual generation), cleaning and validation, and documentation of intended uses and limitations. Although the resulting test set is small, it constitutes a carefully curated test suite for a critical energy decision-support application in an industrial plant.

Based on informal interviews with the cheese plant operators and the analysis of the energy flows described in the previous subsection, we identified seven families of queries that recur in daily operation and energy analysis of the plant. These families are:

- **Key lookup query**: point lookups of information, typically focused on a specific machine, variable, or timestamp. Examples include questions such as “What was the hourly consumption of the milking machine yesterday at 10:00?” or “Give me the latest value of the battery state of charge.” In InfluxDB, these queries are translated into filters over tags and/or specific fields and, in some cases, into the selection of the most recent point.
- **Range query**: queries that explore the behavior of one or several signals over a bounded time interval, without explicit aggregation. For example: “Show me the total active power of the plant between 08:00 and 14:00 last Monday” or “Give me the consumption of chamber 1 over the last weekend”. These queries materialize as time-range filters and, optionally, filters by device or phase.
- **Aggregate query**: queries that require simple aggregations over a time interval, such as sums, averages, maxima, or minima. Typical questions are “What was the total daily consumption of the plant last week?” or “What has been the average PV power today?” In InfluxDB, they correspond to global aggregation functions or basic time-window aggregations.
- **Time sensitive aggregate query**: queries in which the temporal dimension is an explicit part of the analytical requirement, for example, comparisons between periods, sliding windows, or aggregations aligned with work shifts. Examples include “Compare the consumption of the milking machine in the morning shift this week with that of last week” or “Compute the daily self-consumption over the last 30 days and highlight the days with the lowest value.” These queries combine aggregation functions with relative time expressions (e.g., now() - 7d) and alignments to operational windows (shifts, working days, and weekends).
- **Advanced aggregation query**: queries that require more complex or derived aggregations, such as ratios, key performance indicators (KPIs), or multiple grouping levels. Typical examples in the plant are “Compute the self-consumption ratio and the grid-export ratio per day” or “Obtain the ranking of the most energy-intensive machines over the last month.” These queries usually involve aggregations over multiple series, normalization by other quantities, and grouping by tags (machine, phase, and bucket).
- **Join query**: queries that combine information from different buckets or measurements, for example, to cross machine-level consumption with PV production or with hourly predictions. An example is “Compare PV production with the total plant consumption over the last week” or “Relate the consumption of chamber 2 with the battery state of charge.” In Flux, this is implemented through join operations between data streams; in InfluxQL, through subqueries or equivalent combinations.
- **Prediction query**: queries that explicitly request a future prediction from historical time series, leveraging InfluxDB's native forecasting capabilities (e.g., Holt–Winters). Examples of this type, present in the test set, include: “Predict the total plant power for the next hour based on the last 48 h,” or “Predict the hourly consumption of the milking machine for the next 12 h using Holt–Winters.” In these cases, the Flux and InfluxQL queries include both temporal preprocessing (windowing, aggregation) and the corresponding forecasting function call.

These seven categories cover the spectrum of query needs identified in the plant, from point inspection of values and interval exploration to the computation of KPIs and the generation of predictions to support decisions on battery operation, peak management, and production planning.

The test set was built manually, but explicitly following best practices in dataset design recommended by both software engineering for ML systems and the recent literature on responsible dataset creation. First, we defined the parameters and target population of the test set. The objective was not to train models, but to evaluate, in a controlled manner, the system's ability to translate natural language queries into InfluxQL and Flux in a real industrial setting. In particular, the test set evaluates semantic grounding and schema-aware translation rather than the general linguistic capabilities of the language model. Consequently, we restricted the scope to queries that: (i) are formulated in Spanish, reflecting the language and style used by operators in daily interaction; (ii) refer exclusively to the buckets, measurements, fields, and tags described earlier; and (iii) are aligned with relevant operational decisions (energy balance, self-consumption, machine-level consumption analysis, demand, and production forecasting).

Second, we designed a structured sampling strategy within this population. For each of the seven query types described above, we defined eight independent instances, resulting in a total of 56 queries (7 types × 8 examples per type). Each instance combines:

- a natural language text that acts as the input prompt;
- the label of the query type, used for stratified analysis of results; and
- two output representations: one query in Flux and its equivalent query in InfluxQL.

This stratified design guarantees diversity along several axes: diversity of tasks (point lookup, aggregation, join, and prediction), temporal scales (minutes, hours, and days), and plant entities (global variables, individual machines, flows to/from the grid, and the battery). At the same time, the size of the set is deliberately kept small to allow an exhaustive review of each entry.

Third, we applied an internal validation and auditing process. For each of the 56 entries:

1. We manually verified that the natural language text was unambiguous with respect to intent, time window, and entities involved.
2. We checked that the InfluxQL and Flux queries were syntactically correct and executable on the real InfluxDB instance of the plant, returning results consistent with the high-level description (for example, that a Holt–Winters Prediction_Query operates over the data window and horizon stated in the text).
3. We reviewed the semantic coherence between both representations, ensuring that the InfluxQL and Flux queries answer exactly the same question.
4. We avoided including queries with modeling ambiguities (e.g., references to non-existent fields or units absent from the schema), in order not to confound specification errors with errors from the query generation model.

Finally, we documented the intended use and limitations of the test set. In line with recommendations on transparency and dataset documentation, we state that: (i) the set is used exclusively for evaluation and is not employed in training the language models; (ii) its size is small and focused on a single industrial plant, so results should not be extrapolated indiscriminately to other domains or InfluxDB schemas; and (iii) it covers the seven considered query types in a balanced way, but does not aim to be exhaustive with respect to the full space of possible user questions.

Although applied to a small and highly specialized test set, this approach responds to calls from both the software engineering and dataset-creation communities to treat data work as a central component of the lifecycle of ML-based systems, rather than as an ancillary task.

### 3.2 System architecture and implementation

The proposed system enables users to query time-series data stored in InfluxDB 2.0 through natural language interactions. It automatically translates natural language (NL) questions into executable InfluxQL or Flux queries, executes them on the time series database, and presents the results through a graphical interface.

#### 3.2.1 Design description

**Figure 1:**
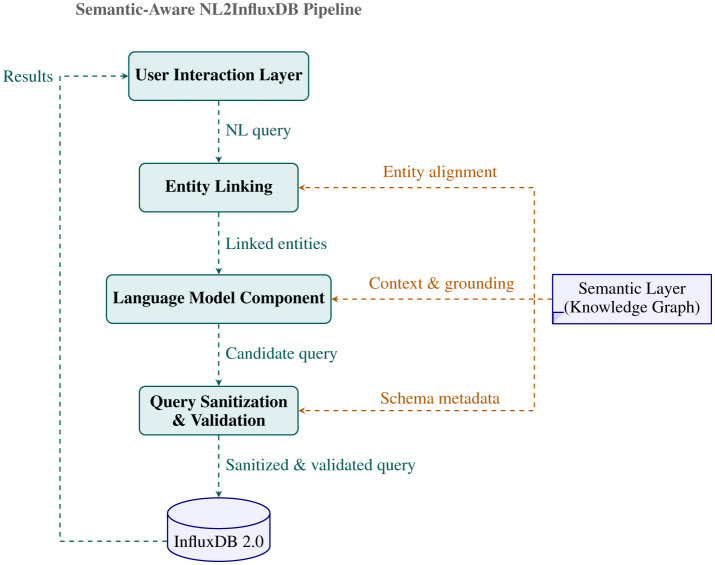
System architecture and component data flow. The Semantic Layer acts as the bridge between the user's natural language query, the Language Model Component, and the InfluxDB schema, ensuring semantic grounding and schema-aware query generation.

The system follows a modular design that separates responsibilities and allows independent evolution of each component. The architecture (illustrated in Figure 1) is composed of five main components:

- **User interaction layer:** Provides a graphical interface where operators enter natural language queries and view the resulting Flux/InfluxQL output and database responses. It acts as the front end of the system and manages user input and result visualization.
- **Semantic layer:** A knowledge graph that encodes the structure and metadata of the InfluxDB schema. It provides the controlled vocabulary used by downstream modules and constrains interpretation to valid buckets, measurements, tags, and fields.
- **Entity linking module:** Bridges natural-language terms and the schema encoded in the Semantic Layer. It identifies relevant buckets, measurements, fields, and tags mentioned in the user query and produces the minimal schema context required by the Language Model Component.
- **Language model component:** A small language model fine-tuned through a two-stage distillation process, combining documentation-based synthetic data generation with model distillation, enabling accurate Flux and InfluxQL query generation from natural language prompts.
- **Query sanitization, validation, and execution module:** Sanitizes generated queries to prevent resource exhaustion and remove unsafe patterns, then verifies their syntactic and semantic correctness before execution against the InfluxDB backend, returning structured results to the user interface.

The modular architecture promotes flexibility and interpretability: the Language Model Component is trained independently, while the Semantic Layer ensures domain alignment and schema awareness.

#### 3.2.2 Semantic layer

This layer provides the conceptual bridge between natural language (NL) user queries and the InfluxDB 2.0 time-series schema. Previous work in NL-to-query systems shows that equipping question-answering systems with KG representation of a database schema significantly improves accuracy in enterprise settings (Sequeda et al., 2024). As highlighted there, graph-based representations preserve the original topology of the database schema, providing a richer contextual representation for each schema element and enabling language models to generate more accurate queries. The benefits attributed in that work to graph-based representations are twofold: they support entity linking against semantically rich descriptions of schema elements, and they expose the topology of the schema—which in general settings includes cross-cutting relations beyond simple containment—directly to the model. The applicability of each of these two contributions depends on the topological complexity of the target schema, an aspect we revisit when describing the InfluxDB schema below.

Aligned with this idea, we use a knowledge graph that formalizes the database's structure and its correspondence with the physical elements of the agro-food production plant. This design enables semantic grounding and structural abstraction, allowing the system to interpret user intent independently of the internal organization of the database.

The graph, modeled under the influx prefix (e.g., influx:Bucket, influx:Measurement, influx:Field, influx:Tag), reuses standard vocabularies such as RDFS and XSD. Nodes represent buckets, measurements, fields, and tags, while relations such as influx:inMeasurement and influx:inBucket express their structural links. The structural backbone of the InfluxDB 2.0 schema is therefore a strict containment hierarchy (bucket ⊃ measurement ⊃ {field, tag}) without cross-cutting relations: in graph-theoretic terms it is a tree rather than a general graph. The relevance of this property is twofold. First, the topological information that the graph exposes can be preserved by any tree-aware serialization, which informs the choice of representation format adopted in the prompt. Second, the semantic richness on which entity linking relies is encoded primarily in node-level descriptions (rdfs:label, dcterms:description, and datatype, enumerations) rather than in the relations themselves. The property influx:hasDataType defines the data type of each field. Textual names from InfluxDB are preserved with rdfs:label, and dcterms:description is used for human-readable metadata, meant to be used by Entity Linking Module.

The descriptions were crafted to maximize semantic separation between all buckets, measurements, tags, and fields, ensuring that each entity in the schema has a unique and unambiguous functional role. They were written using terminology that naturally appears in operator questions while avoiding any form of negative phrasing. For each element, the description highlights the specific physical subsystem, temporal resolution, and analytical purpose it represents, making it easy for a language model to map informal user queries to the correct time-series source. Machine-level elements emphasize individual equipment (e.g., “milking machine,” “cooling chambers”), while plant-level elements emphasize whole-system behavior (solar production, battery SOC, and grid flows). All field descriptions explicitly state their measurement meaning, units, and typical use cases, optimized for robust semantic matching during query generation.

We adopt the RDF Turtle syntax for the KG because it is compact, human-readable and well-suited for inclusion in language model prompts. An excerpt from the case study is shown below:

 
  influx:VRM_hourly_solar_to_cons a
  influx:Field ;
      rdfs:label ‘‘solar_to_cons'' ;
      influx:hasDataType xsd:float ;
      influx:inMeasurement influx:VRM_hourly ;
      dcterms:description
          ‘‘Hourly average power energy delivered
          directly to the
           factory load (kWh). Represents solar
           generation consumed
           on-site in each hour.''
  
  influx:VRM_hourly a influx:Measurement ;
      rdfs:label ‘‘hourly'' ;
      influx:inBucket influx:VRM ;
      dcterms:description
          ‘‘Hourly plant-level dataset including
          total consumption,
          PV production, battery exchange, grid
           import/export,
          and next-day forecasts of consumption,
           PV output,
           and solar irradiance.''
 


Because the semantic layer formalizes domain abstractions independently of database internals, the architectural approach is portable to other TSDB backends that expose comparable semantic structure.

This semantic structure serves three key functions, each operating at a different stage of the pipeline: (1) at the entity-linking stage (Section 3.2.3), it supports disambiguation by mapping user terms to schema entities through retrieval over the enriched node descriptions; (2) at the query-generation stage, it provides the model with a serialized rendering of the relevant schema slice, supplying the structural context required to assemble valid queries; and (3) at the validation stage (Section 3.2.4), it enables semantic consistency checking between the generated query and the actual database schema. The pipeline thus relies on the knowledge graph at three distinct points; the relative empirical contribution of each is examined in Section 4.3.5.

The Semantic Layer provides the controlled vocabulary and structural context required for grounding. To operationalize this link between user queries and the InfluxDB schema, a dedicated entity-linking and subgraph-extraction stage is used. Its design and implementation are described below.

#### 3.2.3 Entity linking and subgraph extraction

To enable the Language Model Component to operate on the correct portion of the schema during inference, the full KG must be reduced to a minimal subgraph that contains only the entities relevant to each user query. This grounding step prevents hallucination and ensures that query generation remains aligned with the actual InfluxDB 2.0 schema.

We implement entity linking as a semantic-retrieval pipeline based on dense embeddings. Each KG resource is encoded not only by its label but by an enriched textual descriptor: bucket descriptors concatenate the names of their child measurements and fields; measurement descriptors combine the names of their fields; field descriptors incorporate human-readable descriptions and datatype annotations; and tag descriptors include enumerated permissible values. This contextual composition ensures that embeddings capture the functional semantics of each entity rather than isolated tokens.

Embeddings are computed using the BAAI/bge-m3 encoder. The user's natural-language query is embedded with the same model, and cosine similarity retrieves the most semantically compatible entities in the KG.

Structural consistency is enforced hierarchically: if a field is selected, its corresponding measurement and bucket are inferred; if a tag is selected, its parent measurement is inferred. When multiple measurements within the same bucket are initially retrieved, they are collapsed into the single best-scoring one. Multiple fields are preserved only when their similarity scores lie within a small relative margin (approximately 2.5%) of the top-scoring field, which allows for multi-field queries while excluding semantically distant candidates.

The result is a minimal, self-consistent schema slice that preserves semantic context while remaining small enough for instruction-following models. This subgraph serves as the schema context supplied to the Language Model Component during query generation.

#### 3.2.4 NL-to-InfluxDB query pipeline

The NL-to-InfluxDB Query Pipeline orchestrates the interaction among the system's core components to transform a natural language input into an executable database query. It establishes a continuous flow from user intent expression to query execution, mediated by semantic grounding through the Knowledge Graph.

As illustrated in Figure 1, the process begins with a question in natural language entered through the user interface, which is then processed by the Disambiguation and Entity Linking module to identify relevant entities within the Semantic Layer. The Language Model Component receives this enriched context and generates queries in InfluxQL or Flux. The Query Sanitization, Validation, and Execution Module first applies a sanitization pass to the generated candidates, patching unsafe patterns such as unbounded time ranges, missing row limits, and unnamed yield() calls, and excluding queries that signal unsupported constructs. The sanitized queries are then validated against the KG schema to verify syntactic correctness and semantic coherence with the underlying InfluxDB schema before being submitted for execution.

Valid queries are executed on the InfluxDB backend and the resulting time-series data are returned to the user interaction layer for visualization.

This architecture externalizes the semantic information required for query generation, allowing the Language Model to interpret the provided schema without relying on internal memorization and enabling adaptation to new schemas without retraining the semantic representation.

### 3.3 Language model development

The development of the Language Model Component constitutes the methodological core of the proposed NL-to-InfluxDB system. This component is trained through a two-stage distillation process that combines documentation-based data distillation and model distillation. In the first stage, knowledge about Flux and InfluxQL query construction is distilled from official InfluxDB documentation using a large teacher model, producing a high-quality synthetic dataset of validated natural language–to–query pairs. In the second stage, this distilled dataset is used to fine-tune a smaller, lightweight model (student), effectively transferring domain-specific knowledge while maintaining computational efficiency.

#### 3.3.1 Synthetic dataset distillation

The dataset distillation pipeline operates through four sequential stages: documentation extraction, contextual retrieval, synthetic sample generation, and multi-layer validation.

Training the Language Model Component requires a corpus of natural language questions paired with their corresponding InfluxQL and Flux queries. Since no public dataset exists for text-to-InfluxQL or text-to-Flux translation, and no available resource captures the structural constraints or query semantics of InfluxDB 2.0, we designed an automated documentation-driven distillation pipeline to construct our training corpus. This choice aligns with recent evidence showing that high-quality synthetic datasets can effectively support model training when expert-annotated data are unavailable. In particular, the GAL framework (Generate–Annotate–Learn) (He et al., 2022) demonstrates that synthetic text can match or even surpass human-labeled data in downstream performance when combined with systematic filtering and validation. Building on these insights, our pipeline generates large volumes of syntactically and semantically validated NL-to-query pairs without human intervention, ensuring both scalability and adherence to the official InfluxDB 2.0 query language specifications.

The source material consists of the official InfluxDB 2.0 documentation for both InfluxQL and Flux, covering query syntax, functions, clauses, and illustrative examples. These documents were downloaded, cleaned, and segmented into semantically coherent units, then indexed in a vector database (ChromaDB) (Chase et al., 2023). During dataset generation, the vector database provides task-relevant contextual snippets for each prompt. This yields a retrieval-augmented prompting scheme, in which relevant documentation fragments constrain the generation process and reduce hallucinations.

Dataset generation is performed iteratively through a large language model (LLM) acting as a *teacher*. In the baseline configuration, a cloud-hosted model (Gemini-2.5 flash) is used.

Each iteration of the process begins with a prompt that includes: (i) a task description specifying:

- the **type** of query to be generated (e.g., Key Lookup, Range, Join, or Time Sensitive Aggregate query) and an explanation for each one.
- the natural–language formulation requirements, ensuring that the generated query description avoids schema elements and reflects domain-level phrasing (the **NL query**)
- the constraints for Flux and InfluxQL translations, and the rule for declaring “*not available”* when a query cannot be expressed in InfluxQL,
- the instructions for producing a concise reasoning trace with three mandatory components (query-type justification, Flux solution rationale, and InfluxQL explanation or limitation) (**reasoning**)
- the requirement to generate an RDF/Turtle schema describing the involved InfluxDB entities (buckets, measurements, fields, and tags), including appropriate use of RDFS, XSD, and optional descriptive vocabularies (**schema**)
- the expected JSONL output structure, including fields such as Query_Type, NL_Query, Flux, InfluxQL, Reasoning, and KG schema,

(ii) the last five queries generated in previous iterations for the same type, which serve as contextual guidance to encourage diversity, and (iii) the content retrieved by the vector database. In each iteration step, the teacher model produces a complete candidate sample in the prescribed JSONL format.

Each JSONL sample then undergoes language-specific consistency checks for both InfluxQL and Flux. The InfluxQL parsing module operates in two main stages. In the first stage, the official InfluxQL parser (InfluxData, 2025b) is invoked to transform the input query into an AST, providing a structured syntactic representation while identifying any parsing errors. Once the query is confirmed to be syntactically valid, a second stage performs semantic validation. In this phase, the AST is integrated with the knowledge graph that encodes the database schema. By aligning the parsed query with this semantic model, the system can detect inconsistencies such as nonexistent fields, incompatible data types, or invalid temporal expressions. This two-layer validation ensures that each query is not only syntactically correct but also semantically consistent with the underlying database structure.

For Flux, the validation process mirrors the InfluxQL workflow: each query is parsed to obtain its AST and then checked for semantic consistency against the knowledge graph. However, it is important to note that Flux is currently in maintenance mode and is not supported by the newer InfluxDB 3, meaning that InfluxQL/SQL is the recommended path for new projects targeting the 3.x ecosystem. In contrast, Flux remains relevant for InfluxDB 1.x/2.x deployments and for maintaining compatibility with existing Flux-based pipelines - as in our case, where the operational system and historical data infrastructure rely on an InfluxDB 2.0 stack that already incorporates Flux queries as part of its monitoring and analytics workflows.

While the InfluxQL parser is publicly available and easily callable as a standalone component, Flux relies on the libflux runtime provided in the official repository (InfluxData, 2025a), accessing its AST required writing a small wrapper around the library. Nonetheless, the resulting validation mechanism operates reliably within our InfluxDB 2.x environment, and the distinction simply reflects the different execution models of the two query languages.

**Figure 2:**
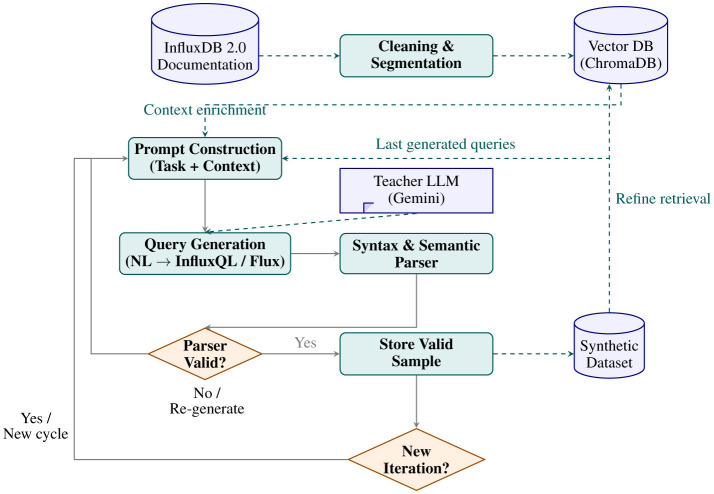
Automated synthetic dataset generation pipeline. The process iteratively distills InfluxQL and Flux query examples from official documentation, using a teacher LLM and an automatic validation loop to ensure syntactic and semantic correctness.

Only validated pairs are stored in the synthetic dataset, while invalid samples trigger corrective regeneration. This fully automated distillation pipeline eliminates the need for manual annotation and ensures alignment with the official InfluxDB query language specifications. Figure 2 summarizes the end-to-end process.

#### 3.3.2 Model pre-training and fine-tuning

To support the development of a lightweight model capable of structured query generation, we adopt a two-stage training pipeline built around the Unsloth framework (Han et al., 2023). Our objective is to maintain the compactness characteristic of SLMs while enabling them to integrate domain-specific knowledge and task-oriented reasoning. This motivates the use of parameter-efficient methods and memory-conscious optimization rather than full fine-tuning of large architectures.

Our training approach follows the fine-tuning (FT) paradigm for LLM-based query generation (Hong et al., 2025), which has proven effective for domain-specific adaptation when paired with high-quality training data and parameter-efficient techniques. Unlike in-context learning approaches that rely on proprietary models and per-query API calls, fine-tuning enables compact models to internalize domain knowledge and query patterns, making them suitable for edge deployment scenarios common in industrial time-series applications. This paradigm aligns with recent advances in data-augmented fine-tuning (Hong et al., 2025), where synthetic corpus generation and task-specific adaptation yield competitive performance with substantially reduced model footprint.

Unsloth provides an optimized environment for adapting language models under tight resource constraints. Its support for 4-bit quantization, fused CUDA kernels, and reduced-memory attention mechanisms significantly reduces the computational footprint of both training and inference. These capabilities align with our aim of refining an SLM without relaxing the hardware requirements typical of small-scale deployments, reflecting the industrial requirement of running inference on constrained hardware environments without dedicated ML acceleration

Unsloth integrates seamlessly with parameter-efficient fine-tuning (PEFT) techniques such as LoRA (Hu et al., 2022). In our setup, LoRA modules serve as the exclusive trainable components added on top of the frozen pretrained backbone. However, unlike classical single-adapter pipelines, our architecture distinguishes between: (i) a domain adapter, used to internalize background knowledge from the InfluxDB ecosystem, and (ii) two language-specific adapters, independently fine-tuned for Flux and InfluxQL. This separation avoids interference between languages while retaining a compact, parameter-efficient design.

##### 3.3.2.1 Stage 1: Domain-level pre-training

This stage focuses on conditioning the model on the InfluxDB documentation over the two query languages, Flux and InfluxQL. The purpose of this step is to expose the SLM to the specialized terminology, constructs, and descriptive patterns characteristic of the InfluxDB ecosystem, without modifying the base model parameters. The LoRA adapter emerging from this stage thus encodes knowledge of the query languages that serve as foundational context during the next task-specific fine-tuning stage.

The resulting adapter acts as an informed initialization for the second training stage. When Stage 1 is enabled, both downstream adapters (Flux and InfluxQL) inherit its weights to ensure that domain-level knowledge propagates consistently into both specialized branches.

##### 3.3.2.2 Stage 2: Task-specific instruction tuning

The second stage specializes the model for the natural language-to-query generation task. Unlike Stage 1, which maintains a single adapter, Stage 2 produces two independent LoRA adapters, one dedicated to Flux and another to InfluxQL. Each training example is routed to the correct adapter depending on its target language, and only the parameters of that adapter are updated.

In short, Stage 1 trains on raw InfluxDB documentation (no NL–query pairs), while Stage 2 trains on the synthetic NL–query dataset; the two stages therefore expose the model to complementary types of information.

The training samples are transformed into a ChatML-style conversational structure to impose consistent instruction-following behavior. The dataset is stratified by query type, and supervised instruction tuning is applied separately for each adapter. If Stage 1 was executed, both adapters are initialized from the shared domain adapter; otherwise, they are initialized directly from the frozen base model.

The dataset is split into training and validation partitions, stratified by query type. Supervised instruction tuning is then applied, again updating only the parameters of the same LoRA adapter initialized in Stage 1. This continuation ensures that domain-level knowledge acquired from the documentation serves as a foundation for learning the procedural, structural, and reasoning requirements of the task-specific dataset.

**Figure 3:**
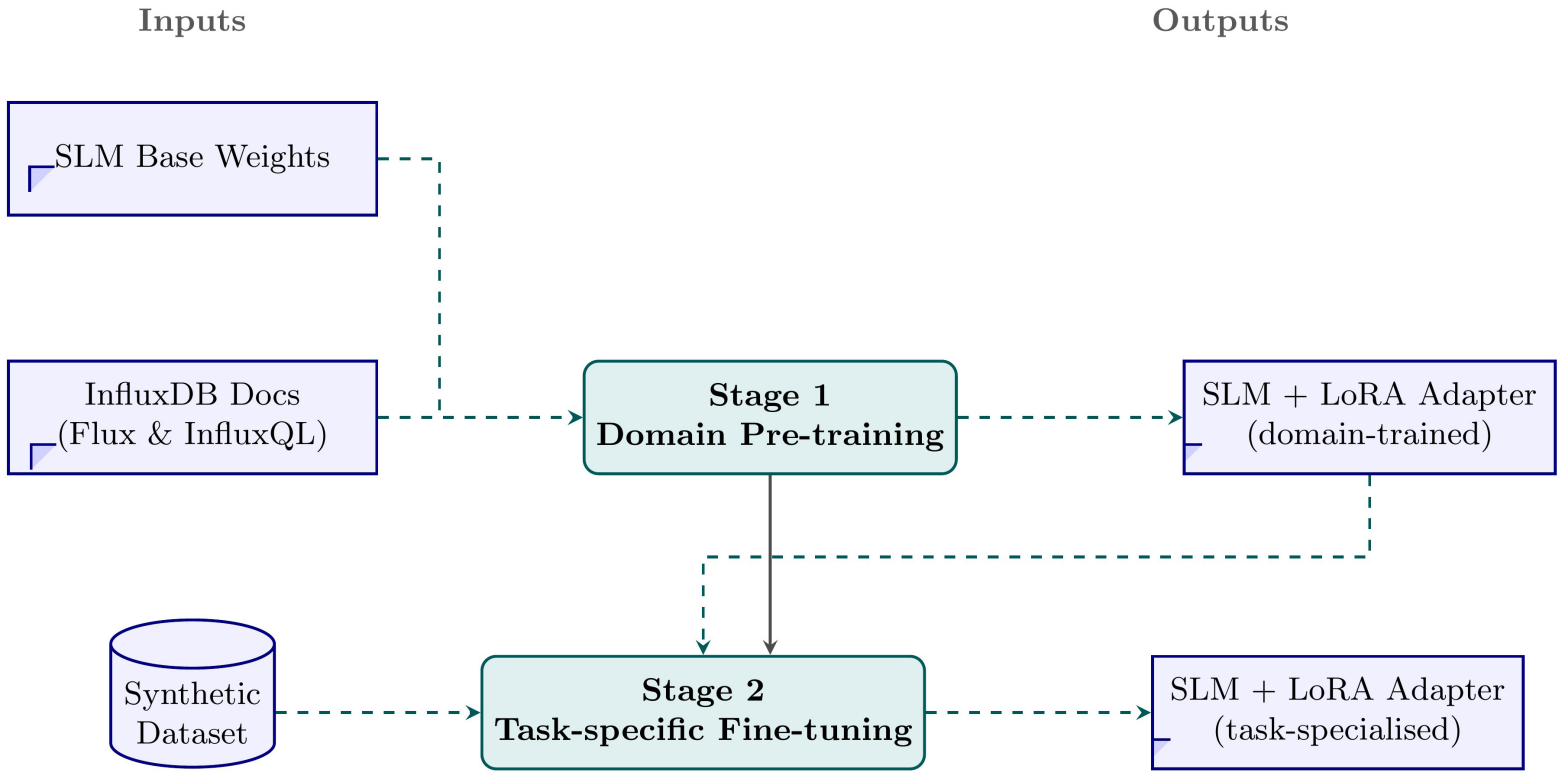
Two-stage training strategy for SLMs. The diagram illustrates the learning flow: Stage 1 performs domain-level pre-training using official InfluxDB documentation to generate a LoRA adapter with contextual domain knowledge; Stage 2 applies instructional fine-tuning using the distilled synthetic dataset to obtain models specialized in generating specific Flux and InfluxQL tasks.

Separating domain conditioning (Stage 1) from task-specific tuning (Stage 2) should allow each stage to introduce a distinct type of information while keeping all updates localized in the same parameter-efficient structure (the LoRA adapter). Figure 3 summarizes this pipeline.

To operationalize this two-stage training pipeline, we draw from the range of lightweight 4-bit models provided by the Unsloth catalog on the HuggingFace Hub. This includes instruction-tuned architectures spanning approximately 270M to 1B parameters, as well as several coder-oriented variants covering the 0.5B–1B range. These models collectively offer a spectrum of trade-offs between compactness, expressive capacity, and GPU memory requirements, ensuring compatibility with hardware-constrained environments while retaining sufficient representational power for the domain- and task-level adaptation performed in our setup. All selected models remain quantized to 4-bit precision and employ the same LoRA adapter across both training stages, preserving the parameter-efficient design that underpins our approach.

## 4 Results

### 4.1 Synthetic dataset

As part of the evaluation of our synthetic dataset generation pipeline, we constructed datasets of 140 queries (20 per predefined query category) using two model variants as teachers (i.e., *Gemini-2.5-Flash-Lite* and *Gemini-2.5-Flash*), across multiple prompting configurations. Each LLM teacher was provided with the five most recently generated queries of the same type as the requested one, ensuring continuity, stylistic consistency, and progressive difficulty across the dataset. While the availability of InfluxDB documentation varied between experiments (either omitted or injected as a 25-chunk, 2,000-character context window), the inclusion of the five-query history was held constant across all experimental conditions. The goal was to evaluate the ability of teacher models to produce the highest-quality synthetic dataset of Flux and InfluxQL queries under controlled prompt-engineering conditions.

Before conducting the full experimental series, the Syntax & Semantic Parser module served as a *feedback mechanism* that enabled iterative improvements to both the prompting strategies and the surrounding orchestration code. By detecting early structural violations, such as invalid operators, unsupported clauses, misaligned tags, or malformed time predicates, the system was refined prior to the large-scale query generation. This incremental feedback loop ensured that the evaluation reflected the intrinsic capabilities of the language models rather than deficiencies in the prompting or execution environment.

#### 4.1.1 Gemini-Flash-Lite-2.5

The Lite model frequently generates *syntactically invalid* InfluxQL, not only through the use of unsupported SQL constructs (e.g., CASE, WHEN, UNION, or timestamp functions such as HOUR()), but also by producing patterns that are fundamentally incompatible with the InfluxQL grammar supported by InfluxDB OSS v2. These include the introduction of explicit or implicit aliases (e.g., AS, or prefixed field references such as s.power_output), incorrect or unsupported join-like structures, misuse of subqueries, and the erroneous use of _time instead of the reserved field time. The model also tends to invent tags or fields not present in the underlying schema. Notably, these structural errors persist *even when detailed domain documentation is injected* into the prompt, indicating that the model does not consistently internalize the syntactic or operational constraints of valid InfluxQL. Consequently, the generated queries systematically diverge from the restricted subset implemented in InfluxDB OSS v2.

In addition to these InfluxQL issues, the *Gemini-2.5-Flash-Lite* model shows recurring problems in the generation of RDF/Turtle schemas. Based on observed outputs, the Lite variant frequently produces Turtle segments containing: (i) repeated or duplicated subjects, and (ii) inconsistent or contradictory namespace declarations. Despite the correctness of accompanying Flux or InfluxQL fields, such malformed Turtle snippets reliably trigger downstream RDF parsing failures.

Because the incorrect Turtle is embedded directly within the JSON output of the model, structurally invalid fragments often propagate into adjacent regions of the object, corrupting the surrounding JSON and complicating extraction, validation, and error attribution. The divergence between the two model variants suggests that the reduced capacity and contextual stability of the Lite model limit its ability to produce mixed-syntax outputs requiring strict adherence to formal grammars, even when schema exemplars and explicit structural constraints are provided in the prompt.

To summarize, the synthetic data experiments establish that parsing-based validation is essential: model capacity alone is not enough to ensure grammatical correctness, and the validation loop improves robustness of the resulting corpus.

#### 4.1.2 Gemini-2.5-Flash

In contrast, *Gemini-2.5-Flash* consistently produces *syntactically well-formed* InfluxQL. The only observed issue was a *semantic* error in duration specification (“invalid duration”), and this occurred *without* any InfluxDB documentation in the prompt context. Notably, Gemini-Flash did *not* generate SQL-like constructs that are invalid in InfluxQL, indicating a stronger implicit representation of the language's grammar. Likewise, Gemini-2.5-Flash did *not* produce malformed CURIEs, duplicate namespace declarations, or corrupted Turtle blocks, suggesting a substantially more stable and internally coherent mastery of RDF/Turtle syntax and graph-structured schema definitions.

#### 4.1.3 Dataset characterization

To characterize the synthetic corpus retained for fine-tuning, we conducted a comparative analysis across three datasets generated with *Gemini-2.5-Flash* under different prompting configurations and sample sizes. The first dataset, Base, consists of 140 queries (20 per category) generated without any injected InfluxDB documentation. The second, DocAug-20, matches Base in size but was produced with a 25-chunk, 2,000-character documentation context window injected into the prompt, following the configuration described above. The third, DocAug-150, extends DocAug-20 with additional samples, reaching 1,050 queries (150 per category), and constitutes the primary corpus used in subsequent experiments. In all three cases, query generation was conditioned on the five most recently generated queries of the same type, ensuring stylistic consistency and progressive difficulty. Token counts for both Flux and InfluxQL representations were computed using the cl100k_base tokenizer from the tiktoken library. Descriptive statistics are reported in Table 1.

**Table 1:** Comparison of synthetic query datasets.

Query type	Flux tokens (mean)			InfluxQL tokens (mean)		
	Base	DocAug-20	DocAug-150	Base	DocAug-20	DocAug-150
Advanced aggregation	468.8	692.4	851.2	2.0	2.0	2.0
Aggregate	116.4	139.9	236.5	28.3	38.6	22.4
Join	938.9	468.6	836.8	2.0	2.0	2.0
Key lookup	137.0	232.0	547.5	61.1	39.2	14.3
Prediction	168.4	136.4	245.0	76.4	64.5	78.9
Range	232.8	217.5	522.8	26.1	18.9	4.3
Time sensitive agg.	925.2	807.9	1743.8	4.0	4.0	2.3
**Overall (mean)**	**426.8**	**384.9**	**712.0**	**28.5**	**24.1**	**18.0**
**Overall (p95)**	**1,327.9**	**1,111.5**	**1,972.3**	**93.1**	**80.2**	**105.0**
*n*	20/20/150 per type			20/20/150 per type		

Base: generated without injected documentation, 20 queries per type; DocAug-20: documentation-augmented prompt, 20 queries per type; DocAug-150: extends DocAug-20 to 150 queries per type. Values are means per query type; overall rows report mean and p95 across all types. Bold values correspond to the two summary rows of the table - Overall (mean) and Overall (p95) – which report the mean and the 95th-percentile token counts aggregated across all seven query types. They are set in bold solely to distinguish these aggregate statistics from the per-query-type values listed above; the bold formatting does not denote optimal, maximal, or otherwise highlighted values.

The three datasets differ substantially in both scale and query complexity. Base and DocAug-20 contain 140 queries each (20 per type), whereas DocAug-150 comprises 1,050 queries (150 per type). Across all datasets, Flux queries are consistently more verbose than their InfluxQL counterparts, reflecting the more explicit, functional nature of the Flux language. The mean Flux token count rises from 384.9 (DocAug-20) to 712.0 (DocAug-150), while the p95 increases from 1,111.5 to 1,972.3, indicating that the larger dataset not only shifts the central tendency upward but also exhibits a heavier tail of structurally complex queries.

The effect of documentation augmentation is most evident when comparing Base and DocAug-20. Despite identical size and type distribution, DocAug-20 yields a lower mean Flux token count (384.9 vs. 426.8), suggesting that documentation guides the model toward more concise and idiomatic Flux expressions. This effect is not uniform across query types: *Join_Query* shows the sharpest reduction (938.9 → 468.6 tokens), consistent with the hypothesis that without reference documentation the model resorts to verbose or non-idiomatic join patterns. Conversely, *Advanced_Aggregation_Query* increases in DocAug-20 (468.8 → 692.4), likely because documentation exposes richer aggregation constructs that the model would not generate from prior knowledge alone.

InfluxQL token counts reflect both the expressive capacity of the language and its documented limitations. Query types with a natural InfluxQL equivalent—such as *Prediction_Query* and *Key_Lookup_Query*—produce non-trivial token counts across all datasets (up to 78.9 and 61.1 tokens respectively). For query types that have no InfluxQL counterpart, specifically *Join_Query* and *Advanced_Aggregation_Query*, the generation pipeline outputs a fixed unavailability marker rather than a query, resulting in the near-constant token count of 2.0 observed across all datasets. This distinction is important for the interpretation of InfluxQL metrics: low token counts in these categories are not indicative of concise expressions but of unsupported functionality, and should be excluded from any cross-language token efficiency analysis.

#### 4.1.4 Discussion

Gemini-2.5-Flash-Lite requires explicit guardrails or post-processing to prevent structural violations of InfluxQL, whereas Gemini-2.5-Flash demonstrates robust syntactic alignment with the language's constraints, even in the absence of external documentation. This contrast highlights significant differences in how both models internalize and generalize domain-specific query languages.

The dataset characterization further supports the conclusion that *Gemini-2.5-Flash* is a suitable teacher model for synthetic query generation. The syntactic validity of the generated queries is a necessary but not sufficient condition for corpus quality; the characterization analysis shows that documentation augmentation also shapes the *semantic* profile of the corpus. The shift from Base to DocAug-20 produces more concise and idiomatic Flux expressions, while DocAug-150 confirms that this generation strategy scales without degradation: the increase in token counts and tail complexity observed in the larger dataset reflects genuine query diversity rather than structural noise. Taken together, these results suggest that combining parsing-based validation with documentation-augmented prompting constitutes an effective pipeline for generating syntactically correct and semantically representative corpora of time-series queries. The inclusion of the most recently generated queries of the same type in the prompt context plays a further role in promoting diversity: by making the model aware of what has already been produced within each category, the pipeline actively discourages repetition and steers successive generations toward broader coverage of measurement contexts, field combinations, and temporal patterns, yielding a corpus that is not only valid but structurally varied.

### 4.2 Model pre-training and fine-tuning

The experiments conducted for SLM model training explore how different training strategies affect the ability of lightweight large language models to generate accurate Flux and InfluxQL queries from natural language instructions and RDF schemas. The study systematically varies two key factors: (1) whether the model undergoes domain-adapted continued pre-training on technical documentation prior to supervised fine-tuning, and (2) whether the supervised fine-tuning includes contextual Chain-of-Thought (CoT) reasoning sequences. These choices yield four training regimes (i.e., pre-training with CoT, pre-training without CoT, no-pre-training with CoT, and no-pre-training without CoT), allowing a controlled investigation of how domain adaptation and reasoning supervision contribute to query generation performance.

The goal of this phase is to systematically evaluate how different SLMs behave under controlled variations of training configuration, using the DocAug-20 synthetic dataset generated with *Gemini-2.5-Flash*, in which the teacher model is supplied with domain-relevant documentation in its prompt context, as described in Section 4.1.3. Each model was fine-tuned under a set of orthogonal configuration switches: (i) the use or omission of chain-of-thought reasoning (CoT = ON/OFF), and (ii) whether an additional domain-level pre-training step (PRE-TRAIN = ON/OFF) was performed before the main instruction-tuning stage.

The models selected for this phase come from the Unsloth project on Hugging Face, which provides memory-optimized instruction-tuned variants of recent SLM architectures (Han et al., 2023). They span two major architectural families—general-purpose instruct models and code-oriented models—represented by Gemma-3 (Gemma Team, 2025), Llama-3.2 (Meta AI, 2024), Granite 4.0 (IBM Research, 2025), and Qwen2.5-Coder (Alibaba Cloud, 2024). To ensure computational comparability, only checkpoints below approximately 1 GB were included.^1^ Whenever feasible, both 4-bit quantized variants and their full-precision counterparts were evaluated, providing a systematic perspective on the trade-offs between memory footprint, expressive capacity, and fine-tuning stability.

Domain pre-training was performed for 25 epochs using a LoRA configuration with *rank* = 64 and α = 64, optimized with a learning rate of 2.5 × 10^−4^. Subsequent instruction fine-tuning employed the same LoRA parameters but was conducted with a reduced learning rate of 5 × 10^−5^. Following standard practice for instruction tuning, prompt tokens were masked from the loss computation so that gradients are computed exclusively over the target query tokens. Fine-tuning followed an early-stopping criterion: optimization terminated once validation loss failed to improve for three consecutive epochs, and the checkpoint exhibiting the lowest validation loss was selected as the final model. Reported results therefore include both the minimum validation loss achieved and the epoch at which it occurred, enabling direct comparison of convergence behavior across model architectures and dataset regimes.

All cross-seed confidence intervals and standard deviations reported in this section are computed over *n* = 3 independent training runs with different random seeds. While this sample size enables detection of gross instabilities and provides directional evidence of configuration effects, it imposes important interpretative constraints. Overlapping confidence intervals between conditions should not be interpreted as definitive evidence of equivalence, and strong claims about relative model ordering are precluded when confidence bounds overlap substantially. The reported intervals reflect estimation uncertainty rather than population-level guarantees, and broader conclusions about architecture families should be drawn cautiously given this limited replication.

#### 4.2.1 Analysis of combined training results

By combining the outputs of the training results into a single table (Table 2), the phase reveals how each model family responds jointly to configuration switches (CoT and PRE-TRAIN) and query language (Flux vs. InfluxQL). To facilitate comparison, the table reports mean best evaluation loss with 95% confidence intervals and mean best epoch across three seeds, enabling patterns of both performance and stability to emerge clearly across architectures.

**Table 2:** Best evaluation teacher forcing loss, per model and configuration: mean with 95% CI (Student-*t*, *n* = 3 seeds) and mean best epoch in parentheses, for Flux and InfluxQL modes.

Model	CoT	PT	*n*	Mean [95% CI] (epoch)	
				Flux	InfluxQL
Gemma3 *270M · 388 MB*	×	×	3	0.1771 [0.1759, 0.1783] (26.0)	0.0376 [0.0374, 0.0378] (17.0)
	×	✓	3	0.1854 [0.1852, 0.1855] (17.7)	0.0397 [0.0378, 0.0416] (11.0)
	✓	×	3	0.1557 **[0.1553, 0.1561] (24.7)**	0.0212 **[0.0209, 0.0214] (15.0)**
	✓	✓	3	0.1642 [0.1641, 0.1644] (18.0)	0.0230 [0.0224, 0.0236] (10.0)
Gemma3 *1B · 1 GB*	×	×	3	0.1356 [0.1352, 0.1359] (20.0)	0.0268 [0.0264, 0.0272] (14.0)
	×	✓	3	0.1407 [0.1397, 0.1418] (13.0)	0.0282 [0.0276, 0.0289] (8.0)
	✓	×	3	0.1177 **[0.1168, 0.1186] (20.0)**	0.0139 **[0.0137, 0.0142] (14.7)**
	✓	✓	3	0.1196 [0.1192, 0.1200] (13.0)	0.0164 [0.0136, 0.0193] (9.3)
Granite4 *350M · 335 MB*	×	×	3	0.2865 **[0.2788, 0.2943] (41.3)**	0.3555 **[0.2715, 0.4394] (48.3)**
	×	✓	3	0.2918 [0.2917, 0.2920] (28.0)	0.4921 [0.4762, 0.5080] (59.3)
	✓	×	3	0.4206 [0.4186, 0.4226] (56.3)	0.6241 [0.6223, 0.6260] (40.0)
	✓	✓	3	0.4260 [0.4257, 0.4264] (34.7)	0.7785 [0.7719, 0.7851] (48.3)
LLaMA3 *1B · 1.03 GB*	×	×	3	0.3397 **[0.3393, 0.3401] (27.0)**	0.3533 **[0.3523, 0.3543] (23.3)**
	×	✓	3	0.3423 [0.3421, 0.3424] (18.3)	0.3578 [0.3577, 0.3580] (16.3)
	✓	×	3	0.4328 [0.4319, 0.4338] (29.0)	0.5623 [0.5619, 0.5627] (23.0)
	✓	✓	3	0.4371 [0.4371, 0.4371] (22.3)	0.5747 [0.5743, 0.5752] (20.0)
Qwen2.5-Coder *0.5B · 457 MB*	×	×	3	0.2200 **[0.2199, 0.2201] (24.0)**	0.2619 **[0.2615, 0.2622] (22.0)**
	×	✓	3	0.2307 [0.2303, 0.2310] (21.0)	0.2772 [0.2762, 0.2781] (19.0)
	✓	×	3	0.3349 [0.3346, 0.3352] (26.0)	0.5117 [0.5110, 0.5123] (25.0)
	✓	✓	3	0.3488 [0.3485, 0.3491] (24.0)	0.5482 [0.5481, 0.5484] (22.0)
Qwen2.5-Coder *1.5B · 1.14 GB*	×	×	3	0.3877 [0.3873, 0.3882] (24.0)	0.3873 **[0.3868, 0.3878] (23.0)**
	×	✓	3	0.3873 **[0.3870, 0.3875] (19.3)**	0.3926 [0.3923, 0.3928] (17.0)
	✓	×	3	0.4559 [0.4558, 0.4560] (26.0)	0.5586 [0.5583, 0.5589] (22.7)
	✓	✓	3	0.4584 [0.4578, 0.4590] (22.0)	0.5628 [0.5623, 0.5633] (20.0)

**CoT**: chain-of-thought; **PT**: domain pre-training (✓ = on, × = off). Model column shows parameter count and 4-bit quantized on-disk size beneath each model name. **Bold**: best configuration per model and mode (lower is better).

Inspection of the combined outcomes highlights three consistent trends across the architectures evaluated:

First, the Gemma-3 family achieves the most favorable convergence minima across both Flux and InfluxQL, with Gemma3 (1B) reaching the lowest overall losses (CoT = ON, PT = OFF: 0.1177 [0.1168, 0.1186] for Flux and 0.0139 [0.0137, 0.0142] for InfluxQL). Confidence intervals are uniformly narrow across all Gemma configurations, reflecting stable convergence across seeds. A consistent pattern across architectures is that Flux requires more epochs than InfluxQL to reach the validation minimum: for Gemma3 (1B), best Flux checkpoints occur at epoch 13–20 depending on configuration, while InfluxQL minima are reached at epoch 8–14, suggesting that Flux generation presents a harder optimization landscape under these training conditions.

Second, domain-adapted pre-training contributes to stability, but its impact is architecture-dependent. The Gemma models benefit modestly from pre-training, yet still achieve competitive minima when trained directly on instruction data. In contrast, Granite4 (0.35B) relies more strongly on pre-training to obtain consistent convergence, especially in Flux. Table 2 shows that Granite PT = OFF configurations exhibit both the widest confidence intervals in the table (InfluxQL CoT = OFF: 0.3555 [0.2715, 0.4394], interval width 0.168) and the highest Flux epoch counts of any architecture (41.3 for CoT = OFF, 56.3 for CoT = ON), indicating slow and unstable convergence without domain adaptation. Pre-training reduces Flux epoch counts markedly (to 28.0 and 34.7) while narrowing confidence intervals across both query languages, converting an unreliable search into reproducible convergence. Granite also shows the clearest Flux–InfluxQL asymmetry in epoch count: PT = OFF requires substantially more epochs for Flux than InfluxQL across both CoT settings, consistent with Flux being the harder optimization target noted above. The InfluxQL pre-training trajectory (Table 3) exhibits a more severe instability: after an initial descent to 1.01 at epoch 11.55, the loss diverges sharply, peaking at 8.51 (epoch 19.24) before partially recovering to 6.76 at the final checkpoint—still 38 × higher than the corresponding Flux final loss. This divergence indicates that Granite's architectural capacity at the 350M scale is insufficient to stably internalize the InfluxQL documentation corpus under the current pre-training configuration. Consequently, enabling pre-training for Granite InfluxQL fine-tuning degrades rather than improves downstream performance: Table 2 shows that PT = ON increases the best evaluation loss by +0.14 (CoT = OFF) and +0.15 (CoT = ON) relative to PT = OFF, the opposite effect observed for Flux, where PT = ON stabilizes convergence. The corrupted domain adapter from the diverged pre-training undermines the fine-tuning stage. Meanwhile, Qwen2.5-Coder (0.5B) shows a pronounced dependence on pre-training, with losses increasing sharply when it is omitted—regardless of whether Chain-of-Thought is enabled.

**Table 3:** Pre-training losses across architectures for the InfluxQL configuration, reported at five intermediate epochs and at the final checkpoint.

Model/epoch	3.87	7.71	11.55	15.39	19.24	23.08
Gemma3 1B	2.765	0.429	0.119	0.038	0.022	0.020
Qwen2.5-Coder 0.5B	1.807	0.372	0.128	0.063	0.029	0.020
Gemma3 270M	2.705	0.737	0.411	0.191	0.085	0.049
LlaMA3 1B	2.157	0.616	0.292	0.210	0.194	0.193
Qwen2.5-Coder 1.5B	3.465	0.840	0.597	0.519	0.498	0.498
Granite4 350M	5.179	1.167	1.012	3.695	8.511	6.763

Domain-adapted continued pre-training on the InfluxDB documentation corpus, LoRA *rank* = 64, α = 64, lr = 2.5 × 10^−4^, 25 epochs, single seed. Rows ordered by final-epoch loss (ascending).

Third, the influence of Chain-of-Thought (CoT) supervision is limited and inconsistent across model families. In Gemma and Granite models, CoT rarely improves final loss values and can even produce slight regressions. The effect is weakest in the Qwen models, which exhibit large variability independent of CoT.

A key observation across the Qwen variants concerns their sensitivity to learning rate. The conservative rate of 5 × 10^−5^ allows optimization to proceed for 19–26 epochs for Qwen2.5-Coder (1.5B) and 21–26 epochs for Qwen2.5-Coder (0.5B) before early stopping triggers, reaching final losses in the 0.39–0.46 and 0.22–0.35 ranges respectively (Table 2). A further distinctive feature is that pre-training does not consistently reduce loss for Qwen: Qwen2.5-Coder (0.5B) reaches its best Flux loss without pre-training (CoT = OFF PT = OFF: 0.220 [0.2199, 0.2201] vs. CoT = OFF PT = ON: 0.231 [0.2303, 0.2310]), and the same pattern holds for InfluxQL. This contrasts with Granite and LLaMA3 (1B), where PT = ON is uniformly beneficial, and suggests that the Qwen-Coder models already encode sufficient domain-relevant priors from pretraining on code corpora to make additional domain adaptation redundant at this dataset scale.

#### 4.2.2 Analysis of inference performance across architectures

Before reporting results, it is necessary to clarify the choice of evaluation metric. For InfluxQL, exact match (EM) is a meaningful criterion because queries are short and structurally constrained, making character-level identity achievable. For Flux, exact match is effectively zero across all configurations: Flux queries are substantially longer, involve numeric range expressions, and admit multiple syntactically valid formulations for the same intent, making literal string equality an overly strict and uninformative criterion. Token F1 (TF1) is used instead as the primary Flux metric. Given a generated query $\hat{q}$ and a reference query q^*^, let Ŵ and W^*^ denote their respective sets of whitespace-delimited tokens (without multiplicity). Precision and recall are defined over the token overlap, as shown in Equation 1:


$$\begin{array}{l} P=\frac{\left|Ŵ\cap W^{*}\right|}{\left|Ŵ\right|},\;R=\frac{\left|Ŵ\cap W^{*}\right|}{\left|W^{*}\right|},\text{ TF}1=\frac{2PR}{P+R} \end{array}$$ (1)

This formulation rewards partial structural overlap—correct function names, field references, and time range tokens—while remaining robust to paraphrastic variation in query structure and insensitive to token repetition. TF1 is reported as the mean over all evaluation examples.

The inference results reported in Table 4 reveal broadly consistent tendencies to those observed in the training evaluation, while offering a more direct view of each model's ability to generate executable queries. Token F1 is used as the primary metric for Flux, where exact match is effectively zero across all configurations due to the complexity and length of Flux queries; for InfluxQL, both exact match (EM) and Token F1 are reported.

**Table 4:** Inference metrics per model and configuration on the held-out split of DocAug-20: Token F1 (TF1) for Flux, and Exact Match (EM) and Token F1 for InfluxQL, with 95% CI (Student-*t*, *n* = 3, clamped to [0, 1]) where available.

Model	CoT	PT	*n*	Flux	InfluxQL	
				TF1	EM	TF1
Gemma3 *270M · 388 MB*	×	×	3	0.135 [0.051, 0.220]	0.667 **[0.381, 0.952]**	0.779 **[0.501, 1.000]**
	×	✓	3	0.214 **[0.190, 0.239]**	0.464 [0.376, 0.553]	0.639 [0.556, 0.723]
	✓	×	3	0.055 [0.014, 0.097]	0.000 [0.000, 0.000]	0.060 [0.000, 0.170]
	✓	✓	3	0.117 [0.073, 0.162]	0.000 [0.000, 0.000]	0.149 [0.091, 0.207]
Gemma3 *1B · 1 GB*	×	×	3	0.229 [0.181, 0.277]	0.619 **[0.219, 1.000]**	0.778 **[0.391, 1.000]**
	×	✓	3	0.259 **[0.238, 0.280]**	0.083 [0.000, 0.268]	0.613 [0.416, 0.810]
	✓	×	3	0.219 [0.179, 0.258]	0.083 [0.000, 0.268]	0.224 [0.055, 0.393]
	✓	✓	3	0.104 [0.079, 0.129]	0.012 [0.000, 0.063]	0.195 [0.163, 0.227]
Granite4 *350M · 335 MB*	×	×	3	0.260 **[0.220, 0.301]**	0.667 **[0.229, 1.000]**	0.763 [0.313, 1.000]
	×	✓	3	0.258 [0.211, 0.305]	0.655 [0.319, 0.991]	0.815 **[0.537, 1.000]**
	✓	×	3	0.158 [0.111, 0.204]	0.000 [0.000, 0.000]	0.075 [0.000, 0.151]
	✓	✓	3	0.123 [0.031, 0.215]	0.000 [0.000, 0.000]	0.067 [0.062, 0.072]
LLaMA3 *1B · 1.03 GB*	×	×	3	0.284 [0.233, 0.335]	0.631 [0.346, 0.916]	0.793 [0.539, 1.000]
	×	✓	3	0.295 **[0.271, 0.319]**	0.679 **[0.525, 0.832]**	0.831 **[0.560, 1.000]**
	✓	×	3	0.259 [0.225, 0.293]	0.000 [0.000, 0.000]	0.175 [0.138, 0.212]
	✓	✓	3	0.182 [0.161, 0.202]	0.000 [0.000, 0.000]	0.105 [0.029, 0.181]
Qwen2.5-Coder *0.5B · 457 MB*	×	×	3	0.295 [0.267, 0.323]	0.667 **[0.381, 0.952]**	0.837 **[0.563, 1.000]**
	×	✓	3	0.309 **[0.283, 0.336]**	0.619 [0.363, 0.875]	0.816 [0.558, 1.000]
	✓	×	3	0.192 [0.139, 0.245]	0.000 [0.000, 0.000]	0.117 [0.110, 0.123]
	✓	✓	3	0.271 [0.244, 0.298]	0.000 [0.000, 0.000]	0.156 [0.123, 0.189]
Qwen2.5-Coder *1.5B · 1.14 GB*	×	×	3	0.354 **[0.299, 0.409]**	0.714 **[0.480, 0.949]**	0.855 [0.640, 1.000]
	×	✓	3	0.319 [0.300, 0.339]	0.702 [0.567, 0.838]	0.861 **[0.625, 1.000]**
	✓	×	3	0.111 [0.086, 0.136]	0.000 [0.000, 0.000]	0.186 [0.089, 0.282]
	✓	✓	3	0.146 [0.118, 0.175]	0.024 [0.000, 0.075]	0.186 [0.120, 0.252]

Flux EM is omitted as it is effectively zero across all configurations. **CoT**: chain-of-thought; **PT**: domain pre-training (✓ = on, × = off). **Bold**: best configuration per model and mode (higher is better).

Three patterns emerge. **First**, Gemma-3 again exhibits strong and stable performance, particularly in the InfluxQL regime. Gemma3 (1B) and Gemma3 (0.27B) both achieve their best InfluxQL results under the CoT = OFF, PT = OFF configuration, with Gemma3 (1B) reaching EM = 0.619 [0.219, 1.000] and TF1 = 0.778 [0.391, 1.000], and Gemma3 (0.27B) reaching EM = 0.667 [0.381, 0.952] and TF1 = 0.779 [0.501, 1.000], consistent with the favorable convergence minima observed during training. A notable dissociation arises when comparing evaluation loss and inference quality with respect to CoT: Table 2 shows that CoT = ON yields lower evaluation losses for both Gemma models (e.g., Gemma3 (1B) CoT = ON, PT = OFF: 0.1177 vs. CoT = OFF, PT = OFF: 0.1356), yet CoT = ON consistently degrades Token F1 and EM at inference time. This indicates that evaluation loss is an insufficient proxy for downstream query generation quality, and that configuration selection should be guided by inference metrics rather than training loss alone.

**Second**, Granite, LLaMA3 (1B), and the Qwen-Coder family display a broadly similar profile in InfluxQL, with best configurations clustered around CoT = OFF regardless of pre-training. Granite achieves its highest TF1 under PT=ON (0.815), while LLaMA3 (1B) reaches its best EM and TF1 also under PT = ON (EM = 0.679 [0.525, 0.832], TF1 = 0.831 [0.560, 1.000]), making pretraining uniformly beneficial for these two architectures in InfluxQL. Among the Qwen models, Qwen2.5-Coder (0.5B) and Qwen2.5-Coder (1.5B) achieve the highest InfluxQL TF1 values in the entire table (0.837 [0.563, 1.000] and 0.861 [0.625, 1.000] respectively), with Qwen2.5-Coder (1.5B) also reaching the highest EM overall (0.714 [0.480, 0.949]). In Flux Token F1, however, no single family dominates: best values across architectures fall in the 0.26–0.37 range and the corresponding 95% confidence intervals overlap substantially, precluding strong claims about relative ordering for this query language under the current dataset and sample size (*n* = 3).

**Third**, CoT supervision is consistently harmful or neutral for InfluxQL performance across all architectures: every model's best InfluxQL configuration has CoT = OFF, and enabling CoT frequently reduces EM to zero. The confidence intervals for CoT = ON configurations are notably narrower than those of CoT = OFF—a pattern that, however, reflects convergence toward uniformly low performance rather than genuine estimation precision. For Flux Token F1, the picture is more nuanced: CoT occasionally produces marginal improvements in individual seeds, but the best Flux TF1 per model invariably occurs under CoT = OFF, and no CoT = ON configuration yields a statistically separable gain given the overlapping confidence intervals. This indicates that chain-of-thought reasoning, as implemented here, does not contribute to structured query generation and in most cases actively disrupts it. These results jointly motivate restricting subsequent experiments to the CoT = OFF regime.

The evidence for domain-adapted pretraining is more nuanced: while its benefit on final Token F1 is modest and architecture-dependent, it plays a critical stabilizing role, particularly for Flux generation. Granite without pretraining exhibits wide cross-seed confidence intervals and high Flux epoch counts without reaching competitive losses (Table 2), while LLaMA3 (1B) shows consistent loss reductions under PT = ON across both query languages. For Qwen, the evidence points in the opposite direction: PT = OFF achieves equal or better validation loss, suggesting that code-oriented pretraining already provides sufficient domain coverage at this dataset scale. Given that PT = ON is necessary for stability in Granite and LLaMA3 (1B), harmful in neither case for Gemma, and only marginally suboptimal for Qwen2.5-Coder (0.5B) (difference < 0.012 in loss), all subsequent experiments in this work are conducted with PT = ON and CoT = OFF.

#### 4.2.3 Analysis of pre-training behavior across architectures

The pre-training trajectories reported in Tables 3, 5 reveal broadly similar tendencies to those observed in the training and inference evaluations, while offering a more granular view of how each model accumulates domain alignment. Three patterns emerge.

**Table 5:** Pre-training losses across architectures for the Flux configuration, reported at five intermediate epochs and at the final checkpoint.

Model/epoch	4.55	9.09	13.65	18.18	22.74
Qwen2.5-Coder 0.5B	1.789	0.248	0.044	0.019	0.013
Gemma3 1B	3.032	0.332	0.056	0.024	0.020
Gemma3 270M	2.924	0.648	0.278	0.094	0.042
Granite4 350M	5.077	1.008	0.607	0.320	0.177
LlaMA3 1B	2.379	0.584	0.321	0.290	0.286
Qwen2.5-Coder 1.5B	3.409	0.790	0.586	0.549	0.546

Domain-adapted continued pre-training on the InfluxDB documentation corpus, LoRA *rank* = 64, α = 64, lr = 2.5 × 10^−4^, 25 epochs, single seed. Rows ordered by final-epoch loss (ascending).

First, Gemma3 (1B) and Qwen2.5-Coder (0.5B) jointly achieve the lowest pre-training losses at the final checkpoint: 0.020 and 0.013 respectively for Flux, and both converge to ≈0.020 for InfluxQL (Tables 3, 5). Gemma3 (1B) descends steadily from 3.032 at epoch 4.55 to 0.020 by epoch 22.74 in Flux; Qwen2.5-Coder (0.5B) shows a sharper early descent (from 1.789 to 0.044 by epoch 13.65) before plateauing at a slightly lower final value. Gemma3 (0.27B) follows a similar trajectory but converges more slowly, reaching 0.042 in Flux and 0.049 in InfluxQL—substantially above Gemma3 (1B) but below all remaining architectures.

Second, Granite-350M and LLaMA3 (1B) remain above Gemma's losses by a significant margin across both query languages. Granite-350M descends steadily without plateauing, reaching 0.177 in Flux at the final checkpoint, but does not approach the minima achieved by the Gemma family. LLaMA3 (1B) converges to a higher final value and shows limited improvement in the later epochs, remaining at 0.286 in Flux and 0.193 in InfluxQL. Qwen2.5-Coder (1.5B) shows the least favorable trajectory: losses remain above 0.54 in Flux and 0.49 in InfluxQL at the final checkpoint with no sign of convergence, confirming that scale within the Qwen-Coder family does not translate to improved domain alignment under pre-training.

Third, the pre-training ranking broadly predicts the fine-tuning ranking reported in Table 2. The models that achieve the lowest pre-training losses—Gemma3 (1B) and Qwen2.5-Coder (0.5B)—also reach the most favorable fine-tuning minima, while Granite-350M and LLaMA3 (1B) occupy intermediate positions in both rankings, and Qwen2.5-Coder (1.5B) remains at the bottom of both. This consistency indicates that pre-training is not correcting architectural weaknesses but rather exposing the inductive alignment of each model with the domain: architectures that absorb domain knowledge efficiently during pre-training also fine-tune more stably. Comparing further with the inference results of Table 4, the correspondence holds for Gemma, Granite, LLaMA3 (1B), and Qwen2.5-Coder (0.5B). The notable exception is Qwen2.5-Coder (1.5B), which records the worst pre-training and fine-tuning losses of all architectures yet achieves the highest InfluxQL Token F1 and exact match in the entire inference table (TF1 = 0.861, EM = 0.714). This dissociation suggests that for this model, instruction fine-tuning on DocAug-20 is sufficient to unlock strong InfluxQL generation independently of domain pre-training alignment, possibly due to the richer linguistic priors accumulated during its larger-scale pre-training on code corpora.

Granite-350M occupies a distinct failure mode: its InfluxQL pre-training diverges catastrophically after epoch 11.55, confirming that not all architectures can stably internalize both query languages under the same pre-training regime. This architectural limitation is reflected in the wide confidence intervals and elevated fine-tuning losses observed in Table 2.

### 4.3 Evaluation on the use case test dataset

Throughout this section, two complementary families of metrics are reported. Lexical and structural metrics—Token F1 (TF1), Exact Match (EM), tokenizer-level cosine similarity, BLEU, ROUGE-L, and parser validity—characterize the surface quality of the generated query and are reported for consistency with prior text-to-query literature. Functional correctness, however, is assessed against the live InfluxDB instance using execution success (exec_ok) and result correctness (result_match), and these are the definitive indicators of operational utility on which model selection for deployment ultimately rests. Evaluation is performed on the held-out test dataset described in Section 3.1.2, comprising 56 queries stratified across the seven query categories, with both Flux and InfluxQL targets.

#### 4.3.1 Selected models

For this evaluation phase, models were selected on the basis of two criteria: strong performance under fine-tuning on DocAug-20 (Section 3.3.2), and the availability of larger-capacity variants within the same architectural family to assess scaling effects. This yields three architectural lines: the Gemma code-oriented line, represented by Gemma3 (1B) and CodeGemma (2B); the Qwen-Coder line, represented by Qwen2.5-Coder (0.5B), Qwen2.5-Coder (1.5B), and Qwen2.5-Coder (3B); and the LLaMA3 line, represented by LLaMA3 (1B) and LLaMA3 (3B), included given their competitive InfluxQL performance in the fine-tuning phase and to assess scaling effects within this general-purpose instruction-tuned family. Additionally, Qwen3-4B is included as an upper-capacity reference representing the most recent Qwen3 instruction-tuned family, providing a ceiling estimate for this model class under the same evaluation conditions.

All models were evaluated under the configuration established in Section 3.3.2: domain-adapted pre-training enabled (PT = ON) and chain-of-thought supervision disabled (CoT = OFF). Models were fine-tuned on the DocAug-150 dataset, which contains 150 synthetic examples per query category (1,050 samples in total), providing broader coverage of use-case patterns while remaining compatible with the resource-constrained training setup adopted throughout this work.

#### 4.3.2 Configuration of the experiments

In contrast with the fine-tuning evaluation phase, where both training and evaluation relied on synthetic natural language instructions paired with minimal schema subsets, this evaluation setting presents a harder and more realistic scenario. Each test instance consists of a natural language query accompanied by a complete knowledge graph describing the database schema, rather than just the subset of entities relevant to the question. Reducing this full graph to a tractable context for SLMs requires an entity-linking step that extracts the minimal subgraph sufficient to interpret the query.

Two evaluation modes are therefore defined. In the *oracle mode*, the model receives the gold minimal subgraph extracted directly from the ground-truth queries, eliminating grounding error and isolating the intrinsic query-generation capability of each model. In the *subgraph mode*, the model receives the reduced schema inferred automatically by the entity-linking pipeline (see Section 3.2.3), reflecting a realistic deployment scenario where grounding is imperfect. The paired design separates the contribution of grounding accuracy from that of generative capacity.

To assess the sensitivity of query generation to the choice of schema representation format, a third evaluation condition is introduced for CodeGemma (2B) in oracle mode. In this condition, the gold minimal subgraph is serialized as YAML rather than RDF/Turtle, keeping all other settings identical (PT = ON, CoT = OFF). This comparison isolates the effect of serialization format from the entity-linking grounding step, which is the primary functional role of the knowledge graph in the pipeline.

#### 4.3.3 Experimental conditions

All experiments were conducted on a single NVIDIA GeForce RTX 4090 (24 GB VRAM) under identical hardware and software conditions. Models were loaded from their Unsloth-optimized 4-bit quantized checkpoints (Han et al., 2023) using the FastLanguageModel inference backend, which applies architecture-specific kernel optimizations, including fused attention, custom CUDA kernels, and graph compilation where supported. Inference was performed using greedy decoding (do_sample = False, temperature = None) and a dynamic max_new_tokens limit set per adapter based on the *p*_95_ reference token length plus a fixed margin, thereby preventing unbounded generation in pathological cases.

Reported inference times ($\bar{t}$ and *t*_95_) correspond to wall-clock measurements per example, including both tokenization and decoding, averaged over the 56 test queries. These times reflect the combined effects of model capacity, quantization level, and Unsloth's architecture-specific optimizations, and should therefore be interpreted as indicative of relative latency within this specific hardware and inference setup, rather than as absolute benchmarks generalizable to other deployment environments. In particular, architectures for which Unsloth provides more mature kernel support (e.g., Gemma, LLaMA) may benefit disproportionately from these optimizations compared to newer or less widely supported architectures.

#### 4.3.4 Analysis across modes and architectures

Tables 6, 7 report the results for each model under oracle and subgraph conditions, respectively. Cross-seed variability at inference time was characterized in Section 3.3.2.

**Table 6:** Oracle mode evaluation results on the held-out test set (56 queries, *n* = 3 seeds).

Model	Mode	Quality				Lat.
		TF1	Sim	BLEU	RL	$\bar{t}$ (s)
Qwen3 *4B · 2.5 GB*	Flux	**0.542 ± 0.01**	**0.646 ± 0.01**	**0.436 ± 0.01**	**0.667 ± 0.01**	9.97 ± 0.1
	InfluxQL	0.542 ± 0.01	0.628 ± 0.01	0.179 ± 0.01	0.607 ± 0.01	5.24 ± 0.2
LLaMA3 *3B · 2.24 GB*	Flux	0.515 ± 0.00	0.598 ± 0.00	0.393 ± 0.00	0.631	6.60
	InfluxQL	0.557 ± 0.04	0.656 ± 0.04	**0.201 ± 0.01**	0.628 ± 0.04	3.56
Qwen2.5-Coder *3B · 2.05 GB*	Flux	0.432 ± 0.14	0.457 ± 0.29	0.306 ± 0.12	0.470 ± 0.30	8.87 ± 0.1
	InfluxQL	0.531 ± 0.05	**0.678 ± 0.08**	0.185 ± 0.04	**0.637 ± 0.09**	4.76
CodeGemma *2B · 2.07 GB*	Flux	0.496 ± 0.02	0.593 ± 0.00	0.374 ± 0.02	0.620 ± 0.01	**1.30 ± 0.1**
	InfluxQL	0.461 ± 0.01	0.571 ± 0.02	0.128 ± 0.00	0.539 ± 0.02	**0.82**
LLaMA3 *1B · 1.03 GB*	Flux	0.441 ± 0.07	0.526 ± 0.07	0.326 ± 0.06	0.560 ± 0.07	4.16
	InfluxQL	0.430 ± 0.02	0.531 ± 0.02	0.122 ± 0.00	0.486 ± 0.03	2.26
Gemma3 *1B · 1 GB*	Flux	0.436 ± 0.01	0.503 ± 0.01	0.307 ± 0.01	0.540 ± 0.01	13.84 ± 1.4
	InfluxQL	0.386 ± 0.04	0.495 ± 0.07	0.093 ± 0.02	0.449 ± 0.07	10.62 ± 4.4
Qwen2.5-Coder *1.5B · 1.14 GB*	Flux	0.115 ± 0.01	0.158 ± 0.01	0.073 ± 0.00	0.143	7.45 ± 0.1
	InfluxQL	0.474 ± 0.05	0.571 ± 0.12	0.162 ± 0.03	0.552 ± 0.10	3.99
Qwen2.5-Coder *0.5B · 457 MB*	Flux	0.309 ± 0.12	0.415 ± 0.14	0.214 ± 0.10	0.427 ± 0.15	6.29
	InfluxQL	0.350 ± 0.09	0.443 ± 0.09	0.079 ± 0.02	0.395 ± 0.08	3.40

Values are mean ± SD. TF1: Token F1; Sim: cosine similarity; RL: ROUGE-L; $\bar{t}$: mean inference time per example. Exact Match is omitted as it is effectively zero across all models and query types. When SD < 0.05 s for latency or < 0.001 for quality metrics, the value is reported without uncertainty. **Bold**: best mean per column per language.

**Table 7:** Subgraph mode evaluation results on the held-out test set (56 queries, *n* = 3 seeds).

Model	Mode	Quality				ΔTF1
		TF1	Sim	BLEU	RL	
Qwen3 *4B · 2.5 GB*	Flux	**0.509 ± 0.01**	**0.611 ± 0.01**	**0.341 ± 0.01**	**0.603 ± 0.01**	−0.033 ± 0.01
	InfluxQL	**0.449 ± 0.02**	0.535 ± 0.02	0.128 ± 0.01	**0.493 ± 0.02**	−0.093 ± 0.02
LLaMA3 *3B · 2.24 GB*	Flux	0.455 ± 0.01	0.537 ± 0.00	0.311 ± 0.00	0.556 ± 0.01	−0.060 ± 0.01
	InfluxQL	0.437 ± 0.02	0.523 ± 0.02	0.118 ± 0.02	0.468 ± 0.02	−0.120 ± 0.02
Qwen2.5-Coder *3B · 2.05 GB*	Flux	0.413 ± 0.13	0.458 ± 0.24	0.273 ± 0.11	0.456 ± 0.24	−0.019 ± 0.13
	InfluxQL	0.429 ± 0.05	**0.542 ± 0.06**	**0.131 ± 0.01**	0.483 ± 0.06	−0.102 ± 0.05
CodeGemma *2B · 2.07 GB*	Flux	0.398 ± 0.02	0.482 ± 0.02	0.259 ± 0.02	0.499 ± 0.02	−0.098 ± 0.02
	InfluxQL	0.419 ± 0.01	0.533 ± 0.01	0.103 ± 0.00	0.476 ± 0.01	−0.042 ± 0.01
LLaMA3 *1B · 1.03 GB*	Flux	0.383 ± 0.05	0.462 ± 0.04	0.254 ± 0.02	0.487 ± 0.03	−0.058 ± 0.05
	InfluxQL	0.399 ± 0.01	0.481 ± 0.02	0.092 ± 0.01	0.411 ± 0.01	−0.031 ± 0.01
Gemma3 *1B · 1 GB*	Flux	0.387 ± 0.01	0.453 ± 0.01	0.249 ± 0.01	0.468 ± 0.01	−0.049 ± 0.01
	InfluxQL	0.364 ± 0.03	0.449 ± 0.05	0.081 ± 0.01	0.389 ± 0.05	−0.022 ± 0.03
Qwen2.5-Coder *1.5B · 1.14 GB*	Flux	0.110 ± 0.07	0.143 ± 0.07	0.067 ± 0.04	0.139 ± 0.08	−0.005 ± 0.07
	InfluxQL	0.421 ± 0.06	0.518 ± 0.13	0.123 ± 0.03	0.470 ± 0.10	−0.053 ± 0.06
Qwen2.5-Coder *0.5B · 457 MB*	Flux	0.268 ± 0.09	0.346 ± 0.12	0.168 ± 0.07	0.351 ± 0.12	−0.041 ± 0.09
	InfluxQL	0.315 ± 0.06	0.393 ± 0.06	0.065 ± 0.00	0.330 ± 0.05	−0.035 ± 0.06

Models receive the automatically inferred minimal subgraph from the entity-linking pipeline. Values are mean ± SD. ΔTF1: difference in Token F1 relative to oracle mode (subgraph minus oracle). TF1: Token F1; Sim: cosine similarity; RL: ROUGE-L. When SD < 0.001 the value is reported without uncertainty. **Bold**: best mean per column per language (TF1, Sim, BLEU, and RL).

##### 4.3.4.1 Oracle mode

In the oracle setting, where grounding ambiguity is eliminated, the ranking across models is broadly consistent across both query languages and aligns with model capacity within each family. For Flux Token F1, Qwen3-4B achieves the highest mean score (0.542), followed by LLaMA3 (3B) (0.515), CodeGemma (2B) (0.496), LLaMA3 (1B) (0.441), Gemma3 (1B) (0.436), Qwen2.5-Coder (3B) (0.432), Qwen2.5-Coder (0.5B) (0.309), and Qwen2.5-Coder (1.5B) (0.115). The anomalous collapse of Qwen2.5-Coder (1.5B) in Flux—observed already in the DocAug-20 evaluation—persists here, confirming it is not a dataset artifact but an architectural incompatibility between this model and Flux generation under the current fine-tuning setup. Qwen2.5-Coder (3B) also exhibits high cross-seed variance for Flux (SD = 0.14), attributable to one run collapsing out of three; its mean masks substantial instability. For InfluxQL, the ordering shifts substantially: LLaMA3 (3B) achieves the highest mean score (TF1 = 0.557), surpassing even Qwen3-4B (0.542), with Qwen2.5-Coder (3B) in third place (0.531), followed by Qwen2.5-Coder (1.5B) (0.474), CodeGemma (2B) (0.461), LLaMA3 (1B) (0.430), Gemma3 (1B) (0.386), and Qwen2.5-Coder (0.5B) (0.350). These results indicate a ranking shift in which the general-purpose instruction-tuned LLaMA3 (3B) outperforms the larger Qwen3-4B in InfluxQL generation despite lower Flux scores, suggesting that InfluxQL's simpler, more SQL-like structure benefits particularly from the broad linguistic priors in LLaMA3's pretraining. Exact Match is effectively zero across all models and both languages, with the limited exception of Qwen2.5-Coder (3B) in InfluxQL (EM = 0.095 ± 0.02) and Qwen3-4B (0.060 ± 0.03), reflecting the high structural complexity of the target queries even under ideal grounding conditions. Flux EM is zero across all models, consistent with the findings reported in Section 3.3.2.

##### 4.3.4.2 Schema representation format

The knowledge graph serves two potential functions in the pipeline: (1) enabling entity-linking to select the correct schema elements from natural language, and (2) providing a semantic graph structure that the model might leverage during query generation. To assess whether the graph structure matters once entity-linking has identified the relevant schema elements, a separate CodeGemma (2B) adapter was trained and evaluated where the entity-linked schema is presented to the model in YAML format—which preserves the same bucket/measurement/field tree as RDF/Turtle but without RDF-specific machinery (namespaces, URI predicates, typed-literal annotations)—rather than RDF/Turtle (a general-graph serialization), keeping all other settings identical (PT = ON, CoT = OFF). Critically, both conditions use the knowledge graph for entity-linking; the difference is only in how the selected schema elements are serialized in the prompt. Table 8 reports results for both formats. For Flux, Token F1 remains unchanged (0.496 under both RDF and YAML), indicating that graph structure does not impact generation quality for the functional, pipeline-oriented query language. For InfluxQL, YAML yields a marginal improvement (TF1 = 0.494 vs. 0.461 with RDF, Δ = +0.033), consistent with YAML's lower lexical overhead: InfluxQL queries are shorter and more pattern-driven than Flux, making them more sensitive to the verbosity of the schema representation in the context window. The near-zero delta for Flux and modest improvement for InfluxQL confirm that once entity-linking has selected the correct schema elements (a function that requires the knowledge graph), the specific serialization in which those elements are presented to the model has minimal impact on generation quality. The functional contribution of the knowledge graph to query generation thus resides in the entity-linking stage—navigating the bucket → measurement → field hierarchy and retrieving against semantically enriched node descriptions—rather than in exposing graph structure to the model at generation time. This finding should be interpreted in light of the structural property of the InfluxDB schema noted in Section 3.2.2: because the schema is a tree, YAML and RDF/Turtle preserve the same topology, and the comparison isolates only the contribution of RDF-specific machinery (namespaces, URI predicates, and general-graph expressivity) on top of that tree, which is shown to be negligible. The contribution of the hierarchical structure itself is not isolated by this ablation, since no strictly flat baseline (e.g., an unstructured list of field identifiers without parent-child nesting) is included; quantifying the marginal value of preserving the tree thus remains open. With this scope clarified, the result supports substituting RDF/Turtle with alternative tree-preserving lightweight representations (JSON, indented key-value pairs) without degrading generation quality, provided the entity-linking step remains intact.

**Table 8:** Schema representation format ablation for CodeGemma (2B) on the held-out test set (56 queries), oracle mode, *n* = 3 seeds, PT = ON, CoT = OFF.

		Flux TF1			InfluxQL TF1		
Mode	Query type	RDF	YAML	Δ	RDF	YAML	Δ
Oracle (gold subgraph)	Adv. Aggregation	0.548 ± 0.03	0.547 ± 0.010	−0.001	0.555 ± 0.05	0.626 ± 0.009	+0.071
	Aggregation	0.603 ± 0.02	0.615 ± 0.007	+0.012	0.617 ± 0.02	0.629 ± 0.066	+0.012
	Join^†^	0.243 ± 0.01	0.233 ± 0.008	−0.010	0.000	0.042 ± 0.072	+0.042
	Key Lookup	0.460 ± 0.02	0.499 ± 0.018	+0.039	0.441 ± 0.03	0.437	−0.004
	Prediction	0.436 ± 0.01	0.429 ± 0.011	−0.007	0.605 ± 0.07	0.615 ± 0.053	+0.010
	Range	0.618 ± 0.02	0.595 ± 0.027	−0.023	0.591 ± 0.04	0.593 ± 0.029	+0.002
	TS Aggregation	0.562 ± 0.03	0.551 ± 0.024	−0.011	0.422 ± 0.03	0.519 ± 0.017	+0.097
	**Overall**	0.496 ± 0.02	0.496 ± 0.007	0.000	0.461 ± 0.01	0.494 ± 0.005	+0.033

**RDF**: standard RDF/Turtle serialization used throughout the paper; **YAML**: hierarchical YAML encoding of the same gold minimal subgraph (preserves the bucket/measurement/field tree with reduced lexical overhead relative to RDF/Turtle). Values are mean ± SD; SD < 0.005 is suppressed. Δ: YAML minus RDF. ^†^Join queries have no valid InfluxQL representation; TF1 measures overlap against the expected “not available” string.

##### 4.3.4.3 Syntactic validity

Table 9 reports the fraction of generated queries that parse without error for each model in oracle mode. Flux syntax accuracy is high across most architectures, with LLaMA3 (3B) achieving perfect syntax (1.000), CodeGemma (2B) and Gemma3 (1B) both reaching 0.994 ± 0.010, Qwen3 (4B) at 0.982, and LLaMA3 (1B) at 0.952 ± 0.021, indicating that fine-tuning is generally sufficient to induce syntactically well-formed Flux output. The two exceptions are Qwen2.5-Coder (3B) (0.708 ± 0.44), whose high variance again reflects the collapsed run, and Qwen2.5-Coder (1.5B) (0.417 ± 0.19), confirming that its Flux failure extends beyond token-level quality to structural validity.

**Table 9:** Syntactic validity of generated queries on the held-out test set in oracle mode (*n* = 3 seeds).

		Flux	InfluxQL	
Model	Size	Syntax acc.	Syntax acc.	N/A
Qwen3	*4B ·2.5 GB*	0.982	0.786 ± 0.036	0.119
LLaMA3	*3B ·2.24 GB*	**1.000**	**0.940 ± 0.057**	0.054
Qwen2.5-Coder	*3B ·2.05 GB*	0.708 ± 0.443	0.708 ± 0.154	0.232
CodeGemma	*2B ·2.07 GB*	0.994 ± 0.010	0.899 ± 0.010	0.000
LLaMA3	*1B ·1.03 GB*	0.952 ± 0.021	0.756 ± 0.135	0.048
Gemma3	*1B ·1 GB*	0.994 ± 0.010	0.661 ± 0.206	0.012
Qwen2.5-Coder	*1.5B ·1.14 GB*	0.417 ± 0.191	0.458 ± 0.477	0.012
Qwen2.5-Coder	*0.5B ·457 MB*	0.804 ± 0.206	0.851 ± 0.150	0.054

**Syntax acc**.: fraction of queries that parse without error, mean ± SD across seeds (SD omitted when all three seeds produced the same value). **N/A**: fraction of InfluxQL queries for which the model produced “not available” rather than a query (averaged across seeds); not applicable to Flux since all query types have a valid Flux representation. **Bold**: highest mean syntax accuracy per language.

For InfluxQL, syntax accuracy is consistently lower across all models, with a drop of 0.10–0.33 points relative to Flux for the same architecture. LLaMA3 (3B) achieves the highest InfluxQL syntax accuracy (0.940 ± 0.057), followed by CodeGemma (2B) (0.899 ± 0.010), which shows the lowest variance among all models. CodeGemma's N/A rate of 0.000 confirms that while it reliably generates syntactically valid InfluxQL queries, it never learns to decline unsupported query types. By contrast, LLaMA3 (3B) combines high syntax accuracy with a modest N/A rate (0.054), indicating a more balanced approach between generating valid queries and recognizing expressibility limits. Qwen2.5-Coder (3B) shows the highest N/A rate (0.232), consistent with its strong Join InfluxQL TF1 score (0.503 ± 0.02), indicating that a substantial fraction of its InfluxQL outputs are deliberate fallback responses rather than failed generation attempts.

##### 4.3.4.4 Subgraph mode

When the model receives the automatically inferred schema subgraph, performance degrades uniformly across all models and metrics, but the degradation is bounded rather than catastrophic. For Flux TF1, the ranking is broadly preserved relative to oracle: Qwen3 (4B) (0.509, Δ = −0.033), LLaMA3 (3B) (0.455, Δ = −0.060), Qwen2.5-Coder (3B) (0.413, Δ = −0.019), CodeGemma (2B) (0.398, Δ = −0.098), Gemma3 (1B) (0.387, Δ = −0.049), LLaMA3 (1B) (0.383, Δ = −0.058), Qwen2.5-Coder (0.5B) (0.268, Δ = −0.041), and Qwen2.5-Coder (1.5B) (0.110, Δ = −0.005). CodeGemma (2B) shows the largest absolute Flux degradation (ΔTF1 = −0.098), suggesting greater sensitivity to schema noise, while Qwen2.5-Coder (3B) degrades minimally (−0.019), indicating robustness to imperfect grounding. For InfluxQL, degradations range from −0.022 to −0.120, with LLaMA3 (3B) showing the largest absolute drop (Δ = −0.120), losing its oracle-mode lead in InfluxQL to Qwen3-4B (TF1 = 0.449 vs. 0.437). Qwen2.5-Coder (3B) also exhibits substantial degradation (−0.102) despite its strong oracle score, suggesting that both models' high oracle performance depends more critically on accurate schema grounding than the code-oriented architectures, which degrade less severely.

##### 4.3.4.5 Scaling within families

Within the QwenCoder family, the scaling trend from 0.5B to 3B is positive for Flux (TF1:0.309 → 0.432 oracle), with Qwen2.5-Coder (1.5B) constituting an anomalous exception. For InfluxQL, the trend is non-monotone: Qwen2.5-Coder (1.5B) achieves higher TF1 than the mean of Qwen2.5-Coder (3B)'s stable runs, suggesting that at this dataset scale the relationship between parameter count and InfluxQL generalization is not straightforward. Within the Gemma family, CodeGemma (2B) consistently outperforms Gemma3 (1B) across both languages and modes (+0.060 Flux oracle, +0.075 InfluxQL oracle), supporting the interpretation that code-oriented pre-training provides a relevant inductive bias for structured query generation independent of parameter count. Qwen3 (4B) achieves the best aggregate results across both languages and modes, with uniquely low cross-seed variance (Flux SD = 0.008, InfluxQL SD = 0.014), indicating that the combination of larger capacity and instruction-following optimization in the Qwen3 family is particularly well suited to this fine-tuning task.

Within the LLaMA3 family, scaling from 1B to 3B yields substantial gains in both query languages: Flux TF1 increases from 0.441 to 0.515 (+0.074) and InfluxQL from 0.430 to 0.557 (+0.127), with the InfluxQL gain being notably larger. This asymmetry suggests that InfluxQL generation—being more SQL-like and closer to the natural language instruction-following tasks in LLaMA3's training distribution—benefits more from increased model capacity than Flux, whose functional, pipeline-based paradigm remains more distant from typical instruction-tuning tasks. The LLaMA3 (3B) results also demonstrate that general-purpose instruction-tuned models can compete with or surpass specialized code-oriented architectures (CodeGemma 2B) on structured query generation, particularly for more declarative query languages, when sufficient capacity is available.

##### 4.3.4.6 Analysis by query type (oracle mode)

Table 10 reports Token F1 broken down by query category for the four best-performing models in oracle mode. Several patterns emerge that are obscured by the aggregate scores.

**Table 10:** Token F1 (TF1) per query type in oracle mode for the four best-performing models, mean ± SD (*n* = 3 seeds).

Mode	Query type	Qwen3 (4B)	Qwen2.5-Coder (3B)	CodeGemma (2B)	LLaMA3 (3B)
		TF1	TF1	TF1	TF1
Flux	Adv. aggregation	**0.590 ± 0.03**	0.497 ± 0.15	0.548 ± 0.03	0.496 ± 0.02
	Aggregation	**0.699 ± 0.01**	0.539 ± 0.11	0.603 ± 0.02	0.626 ± 0.01
	Join^†^	0.257 ± 0.02	0.222 ± 0.07	0.243 ± 0.01	**0.278**
	Key lookup	**0.521**	0.420 ± 0.13	0.460 ± 0.02	0.512 ± 0.02
	Prediction	0.492 ± 0.01	0.400 ± 0.17	0.436 ± 0.01	**0.521**
	Range	0.592 ± 0.03	0.459 ± 0.18	**0.618 ± 0.02**	0.557 ± 0.01
	TS aggregation	**0.643 ± 0.01**	0.485 ± 0.17	0.562 ± 0.03	0.618 ± 0.01
InfluxQL	Adv. aggregation	**0.673 ± 0.03**	0.620 ± 0.05	0.555 ± 0.05	0.621 ± 0.02
	Aggregation	**0.692 ± 0.05**	0.646 ± 0.06	0.617 ± 0.02	0.686 ± 0.01
	Join^†^	0.417 ± 0.19	**0.667 ± 0.14**	0.000	0.292 ± 0.29
	Key lookup	0.453 ± 0.01	**0.503 ± 0.02**	0.441 ± 0.03	0.460 ± 0.05
	Prediction	0.516 ± 0.08	0.471 ± 0.04	**0.605 ± 0.07**	0.596 ± 0.03
	Range	0.567 ± 0.04	0.382 ± 0.26	0.591 ± 0.04	**0.644 ± 0.02**
	TS aggregation	0.475 ± 0.03	0.428 ± 0.24	0.422 ± 0.03	**0.613 ± 0.02**

When SD < 0.005, the value is reported without uncertainty. Bold per row: best mean TF1. ^†^Join queries have no valid InfluxQL representation; the expected output is “not available~.

**Join queries** reveal a fundamental asymmetry between the two languages. For Flux, join-style multi-source queries remain beyond the reach of all four models, with TF1 below 0.26 across the board, confirming that this query category requires compositional reasoning that sub-4B models do not yet generalize. For InfluxQL, join queries have no valid syntactic representation; the expected output is the verbatim string “not available~, so TF1 in this row measures token overlap against that fixed string rather than query generation quality. Qwen2.5-Coder (3B) succeeds most frequently (EM = 0.667 ± 0.14), Qwen3 (4B) partially (0.417 ± 0.19), LLaMA3 (3B) occasionally (0.292 ± 0.29), while CodeGemma (2B) never emits “not available” and instead attempts to generate a query, invariably producing zero token overlap (TF1 = 0.000). This indicates that Qwen2.5-Coder (3B) has learned to associate the join semantic with the expressibility limits of InfluxQL during fine-tuning, a desirable behavior for production deployments where silently generating an incorrect query is more harmful than declining to answer.

**Aggregation queries** (Aggregate, Advanced Aggregation, and Time-Sensitive Aggregate) are where Qwen3 (4B) establishes its clearest advantage in Flux, with TF1 values of 0.699, 0.590, and 0.643 respectively, compared to 0.485–0.618 for the other three models. CodeGemma (2B) performs competitively on standard Aggregation (0.603) and Range (0.618) queries despite its much lower latency, suggesting efficient specialization for the most common query patterns. For InfluxQL aggregations, Qwen2.5-Coder (3B) shows a notable weakness in Time-Sensitive Aggregate (TF1 = 0.428) and Range (0.382) relative to the other models, which depresses its overall InfluxQL score despite its strengths in Aggregation and Advanced Aggregation categories.

**Key lookup and prediction** queries show more uniform behavior across models. CodeGemma (2B) is particularly strong on Prediction InfluxQL (0.605), exceeding Qwen3 (4B) (0.516), and LLaMA3 (3B) (0.596), on this category, consistent with its fast inference profile: prediction queries tend to be more templatic, favoring the compact code-oriented architecture. LLaMA3 (3B) achieves the strongest performance on Range (0.644) and Time-Sensitive Aggregate (0.613) queries in InfluxQL, categories where temporal reasoning and SQL-like filtering operations dominate, aligning with its general-purpose instruction-following strengths.

Taken together, the per-type analysis reveals model-specific strengths across query types and languages. LLaMA3 (3B) emerges as the strongest InfluxQL performer overall, achieving the highest Token F1 (0.557) and leading on Range (0.644) and Time-Sensitive Aggregate (0.613) queries, where SQL-like filtering and temporal reasoning dominate. Its general-purpose instruction-tuning appears particularly well-suited to InfluxQL's declarative structure. CodeGemma (2B) remains the most production-viable choice for latency-sensitive deployments handling standard aggregation and prediction queries, offering roughly 8 × faster inference than LLaMA3 (3B) and Qwen3 (4B) while maintaining competitive accuracy on templatic query types. Qwen2.5-Coder (3B) is the preferred option when InfluxQL fallback recognition for unsupported query types is a priority, emitting “not available” for 66.7 ± 14% of join queries compared to LLaMA3's 29.2%±28.9% and CodeGemma's 0%. Qwen3 (4B) provides the most consistent performance ceiling across query types with the lowest cross-seed variance, though its Flux Prediction catastrophic failure (TF1 = 0.000) and higher latency limit its applicability in production scenarios requiring broad query coverage.

The Token F1 and syntactic validity metrics presented above measure lexical similarity and grammatical correctness but provide limited signal about functional correctness: queries may achieve moderate token overlap or parse successfully yet fail at execution or retrieve incorrect data. To assess end-to-end functional performance, the three best-performing models—CodeGemma (2B), LLaMA3 (3B), and Qwen3 (4B)—were evaluated under live server execution against the InfluxDB instance deployed in the cheese factory.

#### 4.3.5 Server-side execution analysis

Server-side execution was conducted for CodeGemma (2B), LLaMA3 (3B), and Qwen3 (4B) in oracle mode, where each model receives the gold minimal subgraph for every query. The evaluation pipeline records execution success (exec_ok: query executes without error) and semantic correctness (result_match: returned result matches ground truth exactly). A subset of test queries targets time windows for which no data exists in the live instance; queries that execute and return an empty result matching the ground-truth empty result are correctly counted as result_match = True—this is expected behavior, not an evaluation artifact. Additionally, row-level overlap metrics quantify the fraction of returned rows that appear in the ground-truth result, enabling detection of partial correctness beyond binary success/failure.

Prior to server execution, each model-generated query undergoes a sanitization pass to prevent resource exhaustion and server overload—a necessary step given that fine-tuned models occasionally generate unbounded time ranges, computationally expensive aggregations, or unrestricted schema introspection operations. The sanitizer identifies and patches known risk patterns by imposing conservative execution boundaries: a row limit is appended to queries lacking one, Flux queries with unnamed yield() calls are patched to avoid result name conflicts, and queries in which the model explicitly signals that a construct is unavailable in the target language (e.g., join operations in InfluxQL) are excluded from execution entirely via lexical matching. Unlike the syntactic and semantic validation applied during dataset construction (Section 3.3.1), which rejects invalid queries and triggers regeneration, sanitization operates at inference time and modifies rather than rejects queries, ensuring that syntactically valid but semantically unsafe queries do not compromise server availability during automated evaluation.

##### 4.3.5.1 Three-layer validation cascade

The execution metrics expose a systematic three-layer degradation pattern across all models. Parser validity (Table 9) is uniformly high (94%–100% for Flux, 89%–98% for InfluxQL across all models), confirming successful grammar internalization. However, execution success (exec_ok) varies substantially by query type (0.0–1.0), and semantic correctness (result_match) degrades further, typically ranging 0.4–0.7 for the best-performing query types and dropping to near-zero for Time-Sensitive Aggregates. This cascading degradation—high parser validity, moderate exec_ok, low result_match—isolates the distribution gap at the semantic layer: models generate syntactically valid queries that execute without error yet retrieve incorrect data due to misaligned field selection, temporal boundaries, or aggregation logic.

Tables 11–13 report execution metrics for the three models. Time-Sensitive Aggregate queries exhibit the most severe gap across all models: exec_ok remains high (CodeGemma 1.000 Flux/0.917±0.072 InfluxQL; LLaMA3 0.875/1.000; Qwen3 0.792±0.144/0.750) yet result_match collapses to near-zero (CodeGemma 0.000/0.000; LLaMA3 0.333±0.072/0.125; Qwen3 0.042±0.072/0.042±0.072). This indicates that temporal window boundaries are structurally present but numerically incorrect—queries execute successfully but retrieve data from the wrong time range. A contributing factor is that models generate timestamps directly from natural language input without UTC conversion, as timezone information is not represented in the knowledge graph schema. Queries with explicit temporal constraints may retrieve temporally shifted results since underlying data is stored in UTC, partially explaining the zero result_match for this category where sub-hour precision is critical.

**Table 11:** Server-side execution metrics for CodeGemma-2B in oracle mode.

Mode	Query type	exec_ok	result_match	overlap
Flux	Adv. aggregation	0.542 ± 0.144	0.333 ± 0.072	0.600
	Aggregation	0.958 ± 0.072	0.542 ± 0.144	0.722
	Join^†^	0.792 ± 0.072	0.000	0.000
	Key lookup	0.875	0.083 ± 0.072	0.000
	Prediction	0.542 ± 0.260	0.208 ± 0.072	0.467
	Range	0.875 ± 0.217	0.417 ± 0.144	0.896 ± 0.072
	TS aggregation	1.000	0.000	0.000
InfluxQL	Adv. aggregation	0.958 ± 0.072	0.625	0.625
	Aggregation	0.917 ± 0.072	0.375	0.396 ± 0.036
	Join^†^	0.583 ± 0.072	0.000	0.000
	Key lookup	0.958 ± 0.072	0.125	0.125
	Prediction	0.958 ± 0.072	0.375	0.375
	Range	1.000	0.500	0.500
	TS aggregation	0.917 ± 0.072	0.000	0.000

Values are mean ± standard deviation over three runs (*n* = 3). **exec_ok**: query executes without error; **result_match**: returned result matches ground truth exactly; **overlap**: mean fraction of returned rows that appear in ground truth result set (quantifies partial correctness). When SD is zero the value is reported without uncertainty. ^†^Join queries in InfluxQL have no valid representation. CodeGemma-2B never produces “not available” for these queries and instead generates syntactically plausible InfluxQL statements; **exec_ok** reflects the fraction of such generated queries that pass sanitization and execute on the server without error.

**Table 12:** Server-side execution metrics for LLaMA3-3B in oracle mode.

Mode	Query type	exec_ok	result_match	overlap
Flux	Adv. aggregation	0.750	0.500	0.667
	Aggregation	0.875	0.583 ± 0.072	0.667
	Join^†^	0.250	0.000	0.000
	Key lookup	1.000	0.125	0.125
	Prediction	0.542 ± 0.072	0.375	0.750
	Range	1.000	0.708 ± 0.072	0.727 ± 0.034
	TS aggregation	0.875	0.333 ± 0.072	0.381
InfluxQL	Adv. aggregation	1.000	0.750	0.750
	Aggregation	1.000	0.375	0.375
	Join^†^	0.708 ± 0.289	0.000	0.000
	Key lookup	0.875 ± 0.125	0.125	0.145
	Prediction	1.000	0.375	0.375
	Range	1.000	0.583 ± 0.072	0.583
	TS aggregation	1.000	0.125	0.125

Values are mean ± standard deviation over three runs (*n* = 3). Column definitions as in Table 11. ^†^Join queries in InfluxQL: LLaMA3-3B produces “not available” for 0.292 ± 0.29 of join queries (modest fallback recognition); **exec_ok** reflects the fraction of generated (non-N/A) queries that execute without error.

**Table 13:** Server-side execution metrics for Qwen3-4B in oracle mode. Values are mean ± standard deviation over three runs (*n* = 3).

Mode	Query type	exec_ok	result_match	overlap
Flux	Adv. aggregation	0.708 ± 0.072	0.458 ± 0.072	0.645
	Aggregation	1.000	0.542 ± 0.072	0.542
	Join^†^	0.708 ± 0.072	0.000	0.000
	Key lookup	0.833 ± 0.144	0.125	0.153
	Prediction	0.000	0.000	—
	Range	1.000	0.583 ± 0.072	0.583
	TS aggregation	0.792 ± 0.144	0.042 ± 0.072	0.048
InfluxQL	Adv. aggregation	0.875	0.625	0.625
	Aggregation	1.000	0.375	0.375
	Join^†^	0.458 ± 0.191	0.000	0.000
	Key lookup	1.000	0.125	0.125
	Prediction	0.792 ± 0.072	0.375	0.375
	Range	0.833 ± 0.072	0.667 ± 0.072	0.667 ± 0.072
	TS aggregation	0.750	0.042 ± 0.072	0.042 ± 0.072

Column definitions as in Table 11. ^†^Join queries in InfluxQL: Qwen3-4B produces “not available” for 0.417 ± 0.19 of join queries (partial fallback learning); **exec_ok** reflects the fraction of generated queries that execute without error. Notable: Prediction queries in Flux fail completely across all runs (**exec_ok** = 0.000), preventing overlap calculation (—).

Key Lookup queries show similar exec_ok/result_match dissociation but with different semantics: all models achieve high exec_ok (0.75–1.0) yet uniform low result_match (≈0.125), indicating that queries execute without error by retrieving data from plausible measurements but consistently select incorrect fields or tags. This reflects vocabulary mismatch between generic documentation examples and plant-specific schema terminology, compounded by the operational phrasing in test queries vs. the formal structure of training data.

##### 4.3.5.2 Model-specific patterns and failure modes

LLaMA3 (3B) achieves the highest InfluxQL result_match rates across multiple query types: Advanced Aggregation (0.750), Range (0.583 ± 0.072), and Prediction (0.375), consistent with its strong InfluxQL Token F1 reported in Table 6. This advantage extends to exec_ok reliability, where LLaMA3 reaches perfect execution (1.000) for InfluxQL Advanced Aggregation, Range, Prediction, and Time-Sensitive Aggregate queries. However, LLaMA3's Flux performance is more variable: while achieving high parser validity (100%), its Flux result_match for Aggregation (0.583 ± 0.072) is comparable to CodeGemma (0.542 ± 0.144), suggesting that its semantic accuracy advantage is language-specific and may reflect InfluxQL's closer alignment with SQL-like patterns in LLaMA3's instruction-tuning distribution.

CodeGemma (2B) demonstrates strong exec_ok across both languages (Flux: 0.542–1.000 depending on type; InfluxQL: 0.583–1.000), confirming syntactic robustness. Its result_match peaks for InfluxQL Advanced Aggregation (0.625) and shows moderate performance for Aggregation queries in both languages (Flux 0.542 ± 0.144, InfluxQL 0.375). The model exhibits high execution variance for Flux Prediction (±0.260), reflecting run-to-run instability in bucket name generation for forecasting queries. CodeGemma never emits “not available” for InfluxQL join queries (N/A rate 0.000 in Table 9), instead generating syntactically plausible but semantically incorrect statements; this accounts for the partial exec_ok (0.583±0.072) for join queries in Table 11, representing the fraction of invented queries that pass validation and execute without error yet produce no matching result.

Qwen3 (4B) exhibits a catastrophic failure mode unique among all evaluated models: Prediction queries in Flux achieve zero execution success across all three runs (exec_ok = 0.000), indicating systematic generation of invalid bucket references or unsupported forecasting function calls. This complete execution failure is not reflected in Token F1 (Table 10: Qwen3 Flux Prediction TF1 = 0.492 ± 0.01), demonstrating that lexical overlap metrics can assign moderate scores to queries that fail completely at execution—a critical limitation when Token F1 values in the 0.4–0.55 range might be interpreted as indicating partial functional correctness. Conversely, Qwen3 achieves the highest InfluxQL Range result_match of any model (0.667±0.072), outperforming CodeGemma (0.500), and LLaMA3 (0.583±0.072), yet remains below LLaMA3 for Advanced Aggregation (0.625 vs. 0.750). Qwen3 also demonstrates the strongest InfluxQL join fallback behavior, emitting “not available” for 41.7 ± 19.2% of join queries (Table 9), compared to LLaMA3's 5.4% and CodeGemma's 0.000%.

##### 4.3.5.3 Representative failure patterns

Qualitative analysis of generated queries from the evaluation set reveals the specific mechanisms underlying the quantitative failure modes reported above. For Time-Sensitive Aggregate queries, LLaMA3 (3B) frequently generates structurally valid queries that execute without error yet apply incorrect aggregation operators: when asked to calculate energy delta (difference between final and initial readings over a time period), the model generates SUM() instead of the semantically correct LAST() - FIRST() construction, demonstrating comprehension of the query structure and temporal boundaries but failure in operator selection—a canonical instance of the near-miss pattern where high row overlap (queries retrieve data from approximately correct time windows) coexists with zero result_match. In other cases within the same query type, LLaMA3 omits necessary filter predicates (e.g., WHERE name = “ordeñadora” to select a specific equipment tag), retrieving aggregate statistics from the correct measurement and time range but across all equipment rather than the target entity, confirming that the exec_ok/result_match gap can arise from incomplete rather than fundamentally incorrect semantic understanding.

CodeGemma (2B)'s InfluxQL join behavior exemplifies the opposite failure mode: rather than recognizing construct unavailability, the model invents plausible-seeming but nonexistent functions or attempts to reference fields using RDF URI syntax within InfluxQL statements, blending the ontological representation format with the query language and producing queries that fail at parse or execution time. This confirms the zero N/A rate in Table 9: CodeGemma does not emit fallback responses for unsupported constructs but instead generates syntactically creative yet functionally invalid alternatives.

Qwen3 (4B)'s Prediction query failure in Flux provides direct evidence that moderate Token F1 scores can mask complete functional collapse. All eight Prediction queries achieve Token F1 in the 0.45–0.55 range yet fail execution with errors including undefined identifiers and incorrect function arguments (aggregateWindow() called with parameter m instead of interval). The model generates lexically plausible Flux scaffolding—correct from()→range()→filter() pipeline structure—but populates it with invented constructs reflecting pattern-matching on training data without semantic grounding in the Flux standard library. This confirms that lexical overlap metrics, while useful for syntactic fluency assessment, provide insufficient signal for functional correctness evaluation and must be supplemented with execution-based validation to detect semantic failures that preserve surface-level lexical similarity.

##### 4.3.5.4 Partial correctness and near-miss patterns

Row-level overlap analysis reveals that the gap between exec_ok and result_match is not always binary but often gradual, with queries retrieving partially correct result sets. For CodeGemma (2B) Range queries in Flux, row overlap reaches 0.896±0.072 while result_match remains at 0.417 ± 0.144, indicating that approximately 90% of returned rows match the ground truth despite the overall result being marked incorrect. This suggests temporal boundaries are approximately correct but not exact—a “near miss” rather than a fundamental semantic error. Similar patterns emerge for Aggregation queries: CodeGemma achieves overlap 0.722 vs. result_match 0.542 (diff +0.180) in Flux, and LLaMA3 shows overlap 0.667 vs. result_match 0.583 (diff +0.084), indicating partial correctness in field selection or aggregation windows.

In contrast, Key Lookup queries exhibit binary behavior with negligible overlap-result gaps: CodeGemma shows overlap ≈result_match ≈ 0.000, and LLaMA3/Qwen3 both achieve overlap ≈ result_match≈ 0.125. This confirms that field selection is an all-or-nothing operation: queries either select the correct field entirely or fail completely with no partial success. Time-Sensitive Aggregate queries also show minimal overlap despite moderate exec_ok, confirming complete temporal boundary misalignment rather than near-miss behavior. Model-level differences emerge: Qwen3 exhibits more binary behavior overall than CodeGemma or LLaMA3, with overlap closely matching result_match across most query types (differences < 0.01), suggesting less tolerance for approximate answers or a different generation strategy that produces either fully correct or fully incorrect queries with minimal intermediate cases.

##### 4.3.5.5 Distribution gap and semantic grounding

The systematic three-layer degradation and prevalence of near-miss patterns provide quantitative evidence of the distribution gap between documentation-derived synthetic training data and operator-formulated evaluation queries. Models internalize formal grammar successfully (high parser validity) but struggle with semantic mapping (low result_match) despite moderate execution reliability (variable exec_ok). The gap manifests along three dimensions: (1) temporal expression patterns—formal relative references in documentation (“last hour,” “past day”) vs. operational time specifications in plant queries requiring shift-aware or clock-based interpretation; (2) schema vocabulary alignment—generic terminology in documentation examples vs. plant-specific field and tag names in the actual database instance; and (3) contextual grounding—self-contained documentation examples with all information explicit vs. queries with implicit operational assumptions requiring domain knowledge beyond the schema.

The prevalence of near-miss patterns, particularly in temporal queries where overlap can reach 90% despite result mismatch, suggests that models are not fundamentally failing to understand query intent but rather lack the fine-grained semantic annotations needed to bridge vocabulary and temporal precision gaps. This motivates knowledge graph enhancement as a primary direction for future work: adding field aliases, temporal context annotations (shift schedules, operational time windows with approximate match tolerance), and domain-specific terminology mappings could address the semantic layer gap without requiring architectural changes or larger models. The UTC conversion issue represents a concrete, actionable target: incorporating timezone-aware normalization in either the prompt engineering stage or the query sanitization pipeline would address a significant fraction of Time-Sensitive Aggregate failures.

The comparative analysis confirms that no single model dominates across all query types and both languages. Model selection for production deployment must account for the specific query distribution of the target application, tolerance for different failure modes (binary failure vs. partial correctness), and whether syntactic robustness, execution reliability, or semantic correctness is prioritized. CodeGemma (2B) offers the best latency-quality tradeoff with frequent near-miss patterns suggesting room for improvement via semantic grounding enhancements. LLaMA3 (3B) provides the strongest InfluxQL semantic correctness at the cost of higher latency. Qwen3 (4B) delivers the most consistent performance ceiling with lowest cross-seed variance but exhibits the Flux Prediction catastrophic failure and more binary behavior overall, reducing the potential for incremental improvement via schema augmentation strategies.

## 5 Conclusions

This work has presented an end-to-end architecture for natural language interaction with industrial time-series databases, instantiated with real plant data and a production-grade schema from an agro-food facility operating under a self-consumption energy scheme. The system combines an explicit semantic layer implemented as a knowledge graph, a hierarchical entity-linking module, a small language model fine-tuned for NL-to-Flux/InfluxQL generation, and a query sanitization and validation layer. Together, these components allow plant operators to formulate queries in Spanish and obtain executable InfluxDB 2.0 queries without needing to know the underlying schema or query languages.

A central contribution is the documentation-driven synthetic dataset distillation pipeline. By using a teacher LLM with retrieval over the official InfluxDB 2.0 documentation, the system automatically generates (NL, Flux, and InfluxQL) triples that are filtered through parsing, AST inspection, and schema-aware semantic checks. The comparison between Gemini-2.5-Flash and Gemini-2.5-Flash-Lite confirms that both teacher quality and strict validation are crucial to obtain syntactically and semantically reliable training data for domain-specific query languages. The distillation phase demonstrates that large models such as Gemini-2.5-Flash are capable of generating not only syntactically and semantically correct Flux and InfluxQL queries, but also well-formed RDF/Turtle KG schemas describing the involved InfluxDB entities. This confirms the feasibility of the task for sufficiently capable models, and motivates the central contribution of this work: demonstrating that the same query generation capability can be transferred to compact SLMs suitable for resource-constrained industrial deployment through a carefully designed fine-tuning pipeline.

On top of this corpus, we have developed a two-stage, parameter-efficient training strategy for small language models using Unsloth and LoRA: domain-level pre-training on documentation followed by task-specific instruction tuning with separate adapters for Flux and InfluxQL. Experiments across several architectures show that model family and inductive biases matter more than scale alone within the sub-1B regime. Gemma3 models, particularly the 1B variant, consistently exhibit stable convergence and low losses during fine-tuning. Adopting execution success and result correctness as the primary indicators of functional utility, the held-out evaluation reveals a consistent three-layer degradation pattern across all architectures: parser validity is high (98.2%–100% for Flux, 78.6%–94.0% for InfluxQL across the three models evaluated under server execution), execution success (exec_ok) varies substantially by query type (0.0–1.0), and semantic correctness (result_match) degrades further, ranging 0.4–0.7 for the best query types and collapsing toward zero for Time-Sensitive Aggregates. This cascade locates the residual distribution gap at the semantic layer rather than at the syntactic one and is the dominant signal for model selection in deployment. Ranking the three models evaluated under live execution by mean result correctness across the seven query categories, LLaMA3 (3B) leads in both languages (Flux mean result_match≈0.38, InfluxQL ≈0.33), followed by Qwen3-4B (Flux ≈0.25, InfluxQL ≈0.32) and CodeGemma (2B) (Flux ≈0.23, InfluxQL ≈0.29). Lexical metrics partially track this ranking—LLaMA3 (3B) achieves the highest InfluxQL Token F1 (0.557) with perfect Flux syntax validity (1.000) and the best InfluxQL syntax accuracy (0.940), while Qwen3-4B achieves the highest Flux Token F1 (0.542) with the lowest cross-seed variance across both languages—but they do not substitute for it: model selection for deployment should be guided by execution metrics, with lexical scores serving as auxiliary signals of surface fluency. CodeGemma (2B) remains the recommended choice for latency-sensitive deployments where its ~8 × faster inference outweighs the result-correctness gap. A concrete illustration of why execution metrics are necessary appears in Qwen3-4B Flux Prediction queries, where Token F1 reaches 0.492 ± 0.01 while exec_ok collapses to 0.000 across all three runs: lexical-overlap metrics can assign moderate scores to queries that fail completely against the live engine, so conclusions drawn solely from Token F1 in the 0.4–0.55 range may misrepresent functional behavior. Quantized 4-bit versions behave similarly to their full-precision counterparts, indicating that aggressive compression is compatible with accurate NL-to-query generation in this setting and therefore practical for deployment on constrained industrial hardware.

Evaluation on a manually curated test suite tailored to the cheese factory use case demonstrates that the resulting SLMs can reliably generate syntactically correct Flux and InfluxQL queries for a diverse set of operationally relevant tasks. Most evaluated models achieve high syntactic validity in Flux under both oracle and automatically inferred subgraph settings—LLaMA3 (3B) achieves perfect syntax (100%), CodeGemma (2B), and Gemma3 (1B) both reach 99.4%, and Qwen3-4B reaches 98.2%—with the notable exception of Qwen2.5-Coder (1.5B), which exhibits architectural incompatibility with Flux generation under the current fine-tuning setup. The degradation from oracle to inferred subgraphs is moderate, suggesting that the grounding pipeline degrades gracefully under realistic ambiguity. In the InfluxQL setting, models differ in their fallback behavior: Qwen2.5-Coder (3B) and Qwen3 (4B) learn to emit “not available” for query types that InfluxQL cannot express, a desirable behavior for production deployments, while CodeGemma (2B) consistently attempts query generation even for unsupported types. This reflects an architectural difference in how fine-tuning internalizes the expressibility limits of the target language.

Several limitations should be acknowledged. The evaluation dataset is small (56 queries) and focused on a single industrial plant, so results cannot be directly extrapolated to other domains, schemas, or languages. A more fundamental limitation is the distribution gap between synthetic training data and operational queries. The distillation pipeline generates queries from formal technical documentation, which uses standardized terminology and complete temporal specifications. In contrast, real operator queries are conversational, ambiguous, and often underspecified (e.g., “show me yesterday's production” without specifying which measurement, aggregation, or time granularity). The three-layer degradation pattern observed in server execution—high parser validity, moderate exec_ok, low result_match—suggests that models have internalized query syntax and basic semantic structure but struggle with the pragmatic interpretation required to map informal requests to precise database operations. This gap is not linguistic (both training and evaluation queries are in Spanish) but operational: documentation examples are self-contained and explicit, while real queries assume contextual knowledge about plant operations, equipment states, and temporal conventions that are not encoded in the knowledge graph or training data. Large-scale text-to-SQL benchmarks such as BIRD (Li et al., 2023) contain thousands of queries across diverse domains; scaling the current approach to comparable breadth would require substantial expansion of the synthetic distillation corpus and evaluation suite. However, the time-series domain introduces temporal complexities and functional query paradigms (Flux) that extend beyond the relational scope of existing SQL benchmarks.

The synthetic corpora depend on a closed-source teacher model and on the coverage of the official documentation. The current system targets an InfluxDB 2.0 deployment, and the entity-linking component relies on a single embedding model and relatively simple similarity-based selection. In particular, the use of BGE-M3 embeddings (≈560*M* parameters, ≈2.3*GB* RAM footprint) introduces a resource tension: although many language models in the fine-tuning phase operate in the sub-1B regime (457MB–1.7GB in 4-bit quantization), with evaluation extended to 4B parameters (≈3.5*GB* quantized), BGE-M3 represents a deployment overhead comparable to the largest SLMs evaluated, and is therefore not fully aligned with the lightweight-deployment objective for ultra-constrained edge scenarios. Future work should investigate alternative embedding strategies or heuristics that maintain semantic grounding while reducing computational footprint. One promising direction is to complement unified-embedding retrieval with a structured intermediate representation of the user query—effectively turning the raw natural language input into a more canonical form that is easier for the SLM to consume and reason over.

The schema-representation-format ablation isolates the contribution of the knowledge graph along two axes. The grounding step (entity linking against semantically enriched node descriptions) is the dominant contributor to generation quality. The choice between RDF/Turtle and a hierarchical YAML rendering of the same gold subgraph leaves Flux Token F1 unchanged (0.496 under both formats) and yields a marginal InfluxQL improvement (+0.033): RDF-specific machinery as a general-graph format provides no measurable benefit on top of the bucket/measurement/field tree for sub-4B SLMs. Because the InfluxDB schema is itself a tree, this finding rules out the contribution of RDF-specific machinery rather than that of hierarchical structure per se; isolating the latter would require comparison against a strictly flat baseline, which we leave for future work.

Schema linking—mapping natural language tokens to database schema elements—is a central challenge in text-to-SQL systems (Hong et al., 2025). Our entity-linking module addresses this challenge in the time-series domain through knowledge graph-based semantic retrieval with hierarchical structural inference: when a field is selected, its parent measurement and bucket are automatically included, ensuring schema completeness. Future work could adapt advanced schema linking techniques from text-to-SQL, such as learned schema ranking or query-conditional filtering, to the temporal semantics of time-series databases.

Beyond schema linking, the knowledge graph could be enriched with temporal and operational metadata to address current semantic gaps. For instance, augmenting field entities with expected value ranges, sampling frequencies, and operational contexts would enable the system to detect semantically implausible queries even when they are syntactically valid. Similarly, encoding timezone information at the measurement level would support automatic UTC conversion, addressing the temporal boundary misalignment observed in Time-Sensitive Aggregate queries (Section 4.3.5). Such contextual enrichment could reduce the distribution gap between syntactically correct and semantically plausible queries without requiring larger models or more training data.

Future work will also focus on expanding both synthetic and human-authored evaluation datasets across additional plants and query families, and on releasing a public benchmark for NL-to-TSDB queries. We also plan to strengthen semantic grounding with more expressive graph-based reasoning and interactive disambiguation, improve lightweight alternatives to embedding-based linking, reduce reliance on “not available” fallbacks, and extend the architectural pattern to other backends such as InfluxDB 3.x, TimescaleDB, or Prometheus.

Addressing temporal grounding challenges will be critical for improving Time-Sensitive Aggregate query performance. The current system generates timestamps directly from natural language without UTC conversion, leading to temporal boundary misalignment when the underlying data is stored in UTC. Future work should explore: (1) augmenting the knowledge graph with timezone metadata at the measurement or bucket level; (2) developing specialized temporal reasoning modules that infer UTC offsets from conversational context (e.g., “yesterday afternoon” → local time based on plant location → UTC conversion); or (3) fine-tuning the model on temporal examples with explicit UTC annotations to internalize timezone-aware generation. Given that Time-Sensitive Aggregate queries exhibit high exec_ok (0.75–1.0) but near-zero result_match across all models (Section 4.3.5), this represents a high-impact improvement target where relatively small enhancements to temporal grounding could substantially improve functional correctness.

Finally, deployment-oriented studies with real operators will be essential to assess usability, robustness to noisy queries, and the impact of the system on day-to-day energy-management decisions.

## Data Availability

The raw data supporting the conclusions of this article will be made available by the authors, without undue reservation.

## Footnotes

1 The “Size” reflects the on-disk footprint of each model, measured as the size of the model.safetensors file distributed in their official Unsloth repositories.
